# Association of infant formula composition and anthropometry at 4 years: Follow-up of a randomized controlled trial (BeMIM study)

**DOI:** 10.1371/journal.pone.0199859

**Published:** 2018-07-05

**Authors:** Manja Fleddermann, Hans Demmelmair, Christian Hellmuth, Veit Grote, Branka Trisic, Tatjana Nikolic, Berthold Koletzko

**Affiliations:** 1 HiPP GmbH & Co. Vertrieb KG, Pfaffenhofen, Germany; 2 Dr. von Hauner Children’s Hospital, University of Munich Medical Centre, Ludwig-Maximilians-Universität Munich, Munich, Germany; 3 HiPP Study Center, Belgrade, Serbia; 4 Institute for Gynecology and Obstetrics, Clinical Centre of Serbia, Belgrade, Serbia; TNO, NETHERLANDS

## Abstract

The relationships between nutrition, metabolic response, early growth and later body weight have been investigated in human studies. The aim of this follow-up study was to assess the long-term effect of infant feeding on growth and to study whether the infant metabolome at the age of 4 months might predict anthropometry at 4 years of age. The Belgrade-Munich infant milk trial (BeMIM) was a randomized controlled trial in which healthy term infants received either a protein-reduced infant formula (1.89 g protein/100 kcal) containing alpha-lactalbumin enriched whey and long-chain polyunsaturated fatty acids (LC-PUFA), or a standard formula (2.2 g protein/100 kcal) without LC-PUFA, focusing on safety and suitability. Non-randomized breastfed infants were used as a reference group. Of the 259 infants that completed the BeMIM study at the age of 4 months (anthropometry assessment and blood sampling), 187 children participated in a follow-up visit at 4 years of age. Anthropometry including weight, standing height, head circumference, and percent body fat was determined using skinfolds (triceps, subscapular) and bioelectrical impedance analysis. Plasma metabolite concentration, collected in samples at the age of 4 months, was measured using flow-injection tandem mass spectrometry. A linear regression model was applied to estimate the associations between each metabolite and growth with metabolites as an independent variable. At 4 years of age, there were no significant group differences in anthropometry and body composition between formula groups. Six metabolites (Asn, Lys, Met, Phe, Trp, Tyr) measured at 4 months of age were significantly associated with changes in weight-for-age z-score between 1 to 4 months of age and BMI-for-age z-score (Tyr only), after adjustment for feeding group. No correlation was found between measured metabolites and long-term growth (up to 4 years of age). No long-term effects of early growth patterns were shown on anthropometry at 4 years of age. The composition of infant formula influences the metabolic profile and early growth, while long-term programming effects were not observed in this study.

## Introduction

Associations between infant feeding, growth velocities and later life outcomes are known [1,2,3,4]. Rapid weight gain during the first two years of life is associated with an increased risk of obesity later in life, as documented in several studies [2,3,4]. The rate of weight gain in the first two years of life can effectively be modulated by feeding choices during infancy [5]. For example, high protein intake during infancy is linked to an increased risk of rapid early growth as well as later obesity [1,6].

Results of a multicenter, double-blind, randomized clinical trial (European childhood obesity project), in which infants received either a formula with a higher protein content or a reduced protein content during the first year of life [7], show the correlation between high early protein intake, enhanced early and long-term body weight, and associated metabolic patterns. Programming effects of increased levels of amino acids in plasma and tissue [7] and possible correlation with an increased adipogenic activity [8] have been proposed. A saturation of the branched-chain amino acid degradation pathway was observed with a high protein intake during infancy [9]. The European childhood obesity project has indicated that lyso-phosphatidylcholine C14:0 in serum of 6 month old infants is associated with weight gain during infancy and childhood, but the underlying mechanisms are not fully understood [10].

While the influence of protein quantity during early life on later outcomes is reported in several studies, the influence of protein quality on longitudinal changes in body composition is less clear.

The Belgrade-Munich infant milk (BeMIM) trial is a randomized controlled trial that aimed to assess the suitability of a protein-reduced infant formula containing alpha-lactalbumin enriched whey and long-chain polyunsaturated fatty acids (LC-PUFA). The primary evaluation (safety and suitability) of the trial focused on growth velocities, adverse events, markers of fatty acids and protein status in infants up to an age of 4 months. The aim of the follow-up (FU) study, presented here, was to examine the influence of the protein intake between the ages of 1–4 months on anthropometry and body composition at the age of 4 years and to investigate the relationship between nutrition, early growth, long-term growth and metabolic effects. In addition, it was investigated whether the infant metabolome at the age of 4 months might predict anthropometry at 4 years of age (long-term growth) or may explain short-term growth until 4 months of age.

## Materials and methods

### Study design and protocol

Children included in the BeMIM study, a randomized, double-blind, controlled infant feeding trial with parallel design [11], were invited to participate in the follow-up (FU) visit at 4 years of age. In the BeMIM trial, healthy term infants received either a control infant formula (CF, 2.2 g protein/100 kcal) or an intervention infant formula with a lower protein content (IF, 1.89 g protein/100 kcal), but a higher proportional content of alpha-lactalbumin enriched whey, and added LC-PUFA. Both formulae had identical whey/casein ratios of 60/40 and energy contents of 67 kcal/100 mL and were provided to infants until the age of 4 months (intervention period).

Of 213 formula-fed infants that were enrolled during the first month of their life, 167 infants (IF n = 85, CF n = 82) completed the intervention study at 4 months and formed the intention-to-treat population (per protocol: IF n = 46, CF n = 35) [11]. Non- randomized breastfed infants (BF, n = 185) were enrolled as a reference group. A total of 92 BF infants completed the study at 4 months (per protocol: BF n = 54). Children (n = 259) who had completed the trial were invited to participate in the FU study at 4 years of age. The study protocol was approved by the Clinical Center Serbia Ethical Committee, and parents provided their informed consent. The trial was registered at ClinicalTrials.gov (NCT01094080) on February 1, 2010.

Anthropometry including weight, height, head circumference and skinfold measurements (triceps, subscapular) as well as bioelectrical impedance analysis (BIA) were performed by trained study personnel at 4 years of age. The infants’ medical history between 4 months and 4 years of age was assessed based on hospital and parental reports. Weight was determined using a Seca 711 scale (Seca, Hamburg, Germany) equipped with a measuring rod (Seca 220) for measuring height. Head circumference was measured using a tape measure (Seca 212). All measurements were performed in duplicate and documented with an accuracy of 10 g for weight and 0.1 cm for length and head circumference. The equipment was checked every two months using a calibrated weight of 5.00 kg to ensure accuracy of measurements. There was never a deviation of more than 10 g. Z-scores for age were calculated using anthropometric results relative to WHO growth standards [12].

Triceps and subscapular skinfolds were measured using a Holtain caliper (Holtain Ltd, Crymych, UK) at the left body axis. All measurements were performed in triplicate and documented with an accuracy of 0.2 mm. Body fat percentage was calculated via predictive skinfold equations according to Slaughter et al. 1988 [13]. Bioelectrical impedance analysis was performed in duplicate using a BF 906 Body Fat Analyzer (Maltron, Rayleigh, United Kingdom) and resting in a supine position by placing 2 electrodes at standardized points on the right hand and 2 electrodes on the right foot.

### Laboratory analyses

At 4 months of age, venous blood samples were drawn >2 hrs after the last feed. Plasma aliquots were stored at -80°C, then transported on dry ice to the Dr. von Hauner Children’s Hospital (Munich, Germany) where the analyses of phospholipids and acyl-carnitines using flow-injection mass spectrometry according to Uhl et al. 2016 were performed [14]. Plasma amino acid concentrations have been presented before [11,15].

Analytical quality (intra- and inter-batch variation) was evaluated based on the coefficients of variation obtained from repeated analysis of aliquots of two standard plasma samples.

### Statistical analysis

Statistical analyses were performed using Stata®/MP 11.0 (StataCorp LP, College Station, TX, USA). A Pearson’s chi-square test was used for statistical comparison of categorical data.

Analyses on anthropometry and body composition at 4 years of age, as well as growth from birth until 4 years of age, are presented as mean ± standard deviation. Student’s t-test was used for normally distributed continuous variables to estimate the effect of formula type on anthropometric outcome. Linear regression models were applied to estimate the effect of formula type on anthropometric outcome, including children’s age, the respective baseline value at 30 days of age (if available), gender, maternal age and maternal smoking status as adjusted variables. Effect sizes are presented as coefficient and 95% confidence intervals. Significance was accepted at p<0.05.

Metabolomic analyses (carnitine and carnitine ester concentrations) at 4 months of age are presented as medians and interquartile ranges (IQR, 25th and 75th percentile). The Kruskal-Wallis rank test was used for abnormally distributed continuous variables. Linear regression models were applied to estimate the associations between each metabolite and WHO-standardized weight/length/weight-for-length/BMI between birth and 4 years of age, with metabolites as independent variables. Due to the analysis of 129 metabolites, Bonferroni-corrected p-values (0.05/129 = 0.00038) were used for significance evaluation. All analyses were repeated with adjustment for feeding group.

Anthropometry outcomes at 4 years of age were analyzed for intention-to-treat and per-protocol populations. The intention-to-treat analysis included all randomized subjects that received study formula during the intervention period. The per-protocol analysis included only data from subjects complying with predefined conditions during the intervention period: no intake of formula other than study formula, a maximum daily intake of 50 ml tea and less than 3 spoons of complementary food per week. Missing data at individual time points were not replaced.

## Results

### Population

**Fig 1:**
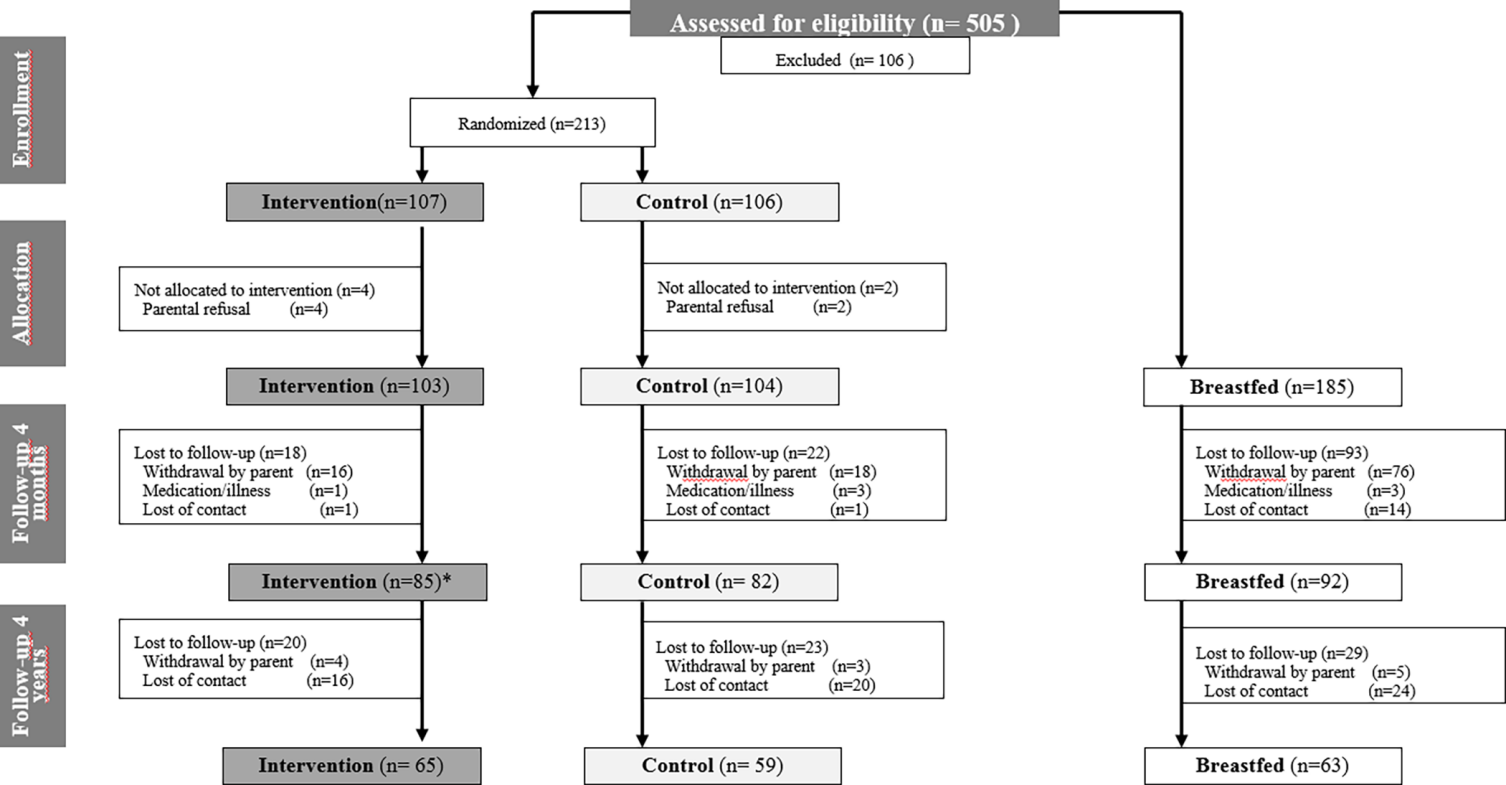
CONSORT chart: Numbers of participants for randomization, allocation, follow-up and analysis. * Of which 3 infants were excluded from analyses.

A total of 259 children (IF: 85, CF: 82, BF: 92 infants) that completed the BeMIM study at 4 months of age were invited to participate in the FU visit. Out of this original study population, 60 children could not be located (IF: 16, CF: 20, BF: 24 infants), and twelve parents (IF: 4, CF: 3, BF: 5 infants) declined the invitation to participate (Fig 1), resulting in a distribution of 65 children in the IF, 59 in the CF and 63 in the BF group who completed the FU study. A total of 42 children in the IF, 25 in the CF and 40 in the BF group were part of the per-protocol population in the FU study. The FU study took place between 21.01.2014 and 08.04.2015. Numbers of drop-outs from the original study population and the reasons for dropping-out did not differ between groups.

The characteristics of the study population are shown in S1 Table. There were no differences between formula groups at 4 years of age. Breastfed children differed from formula-fed children in terms of maternal educational status, which is consistent with observations at baseline (age 1 month). The mean age at follow-up was 3.95 ± 0.03 years, with no difference between randomized formula groups.

Furthermore, the study population at 4 years of age differed slightly from the original study population with respect to age of mothers at delivery and percentage of mothers smoking during pregnancy. The age of mothers at delivery was significantly higher in families that participated in the FU study than in those who did not (31.6 vs. 29.4 years, p = 0.003). The percentage of mothers who smoked during pregnancy was lower in FU participants than in non-participants (21% vs. 35%, p = 0.03). No differences were observed with respect to maternal educational status or parental body mass index (BMI).

### Growth, z-scores and body composition of children up to 4 years of age

**Fig 2:**
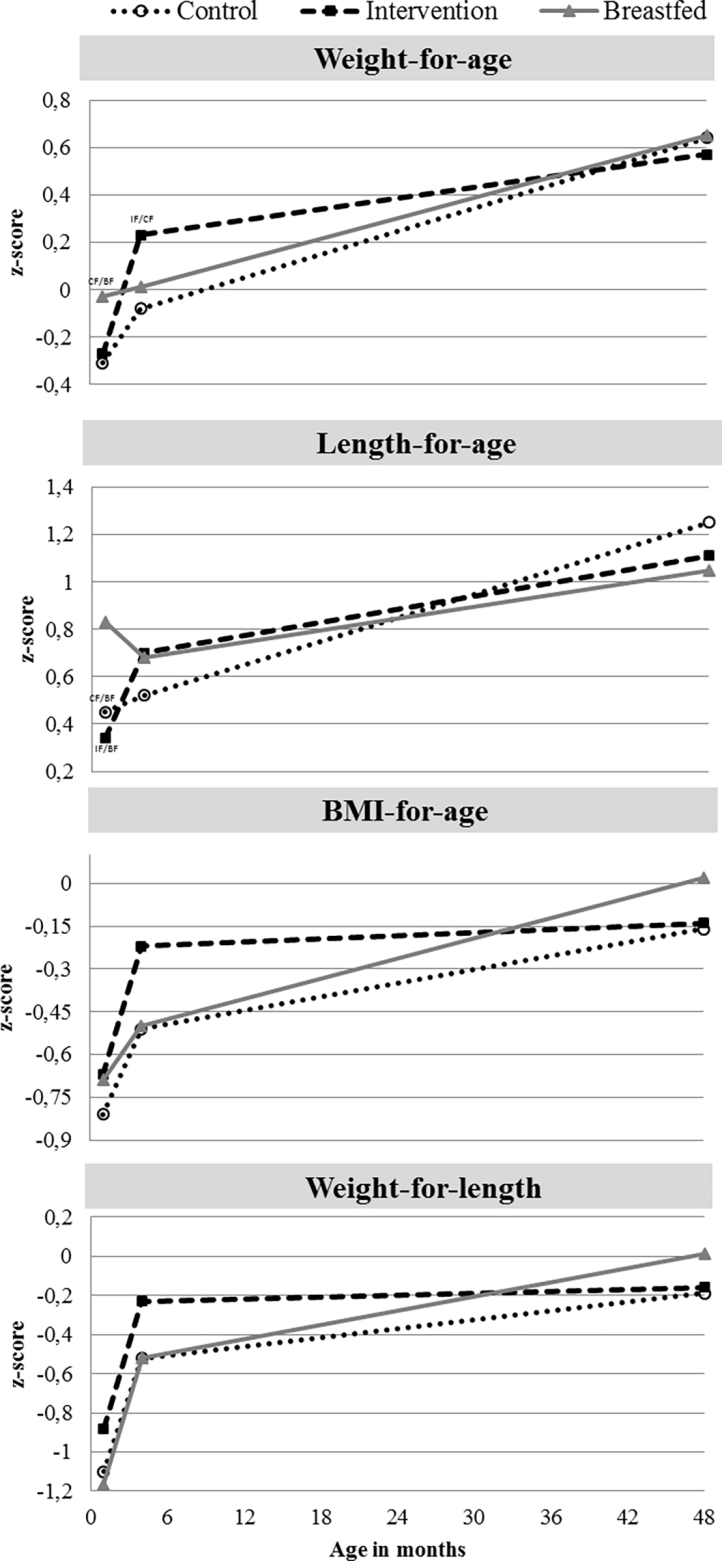
Mean z-scores for weight, length, BMI and weight-for-length in the intervention, control and breastfed groups from 1 month to 4 years of age of the intention-to-treat population (follow-up children only).

Infant growth in the interventional study up to the age of 4 months was published in Fleddermann et al. [11]. Fig 2 presents z-scores (weight-for-age, length-for-age, BMI-for-age, weight-for-length) between 1 month and 4 years of age of children participating in the FU visit. The increase in weight and length z-scores between 1 and 4 months of age was higher for IF than for CF infants (Table 1), while the increase of BMI z-scores did not differ. After 4 months of age a significantly lower increase in z-scores (for weight and length) was observed in the IF compared to the CF group. The adjustment for confounders had no effect on group difference of change of z-score during the first 4 years of life.

**Table 1:** Change of z-scores during the first 4 years.

	Formula group								Estimated difference between IF and CF ^1^	Breastfed group			
	Intervention				Control								
	n	Mean ± SD			N	Mean ± SD				n	Mean ± SD		
**Change in z-score from 1 month to 4 months–all infants**													
WAZ	82	0.51	±	0.68	82	0.29	±	0.77	0.21 (-0.01,0.42);p = 0.06	92	0.08	±	0.75
LAZ	82	0.34	±	0.72	82	0.05	±	0.77	0.21 (0.003,0.42);**p = 0.047**	92	-0.07	±	0.78
BMIZ	82	0.48	±	0.94	82	0.38	±	0.99	0.14 (-0.13,0.41);p = 0.32	92	0.17	±	1.05
WLZ	82	0.66	±	1.15	82	0.70	±	1.07	0.11 (-0.17,0.36);p = 0.44	92	0.61	±	1.17
**Change in z-score from 1 month to 4 months–follow-up children only**													
WAZ	65	0.50	±	0.62	59	0.23	±	0.76	0.29 (0.06,0.52);**p = 0.01**	63	0.05	±	0.69
LAZ	65	0.36	±	0.73	59	0.07	±	0.81	0.25 (0.01,0.48);**p = 0.04**	63	-0.15	±	0.76
BMIZ	65	0.45	±	0.86	59	0.30	±	0.98	0.23 (-0.07,0.52);p = 0.13	63	0.19	±	0.99
WLZ	65	0.65	±	1.06	59	0.58	±	1.10	0.22 (-0.09,0.53);p = 0.16	63	0.65	±	1.11
**Change in z-score from 4 months to 4 years**													
WAZ	65	0.34	±	0.84	59	0.72	±	0.66	-0.37 (-0.64,-0.10);**p = 0.007**	63	0.63	±	0.80
LAZ	65	0.40	±	0.92	59	0.72	±	0.85	-0.33 (-0.65,-0.02);**p = 0.04**	63	0.37	±	0.85
BMIZ	65	0.08	±	1.16	59	0.34	±	0.82	-0.26 (-0.62,0.11);p = 0.17	63	0.51	±	0.98
WLZ	65	0.08	±	1.17	59	0.33	±	0.84	-0.25 (-0.62,0.12);p = 0.18	63	0.53	±	0.98
**Change in z-score from 1 month to 4 years**													
WAZ	65	0.84	±	0.96	59	0.95	±	1.05	-0.08 (-0.40,0.25);p = 0.64	63	0.68	±	0.88
LAZ	65	0.76	±	1.09	59	0.80	±	1.07	-0.09 (-0.42,0.25);p = 0.61	63	0.22	±	0.97
BMIZ	65	0.52	±	1.10	59	0.64	±	1.11	-0.03 (-0.39,0.33);p = 0.88	63	0.70	±	1.22
WLZ	65	0.73	±	1.19	59	0.91	±	1.16	-0.03 (-0.38,0.32);p = 0.87	63	1.18	±	1.35

WAZ, weight-for-age z-score; LAZ, length-for-age z-score; BMIZ, BMI-for-age z-score; WLZ, weight-for-length z-score; IF, intervention formula; CF, control formula. Data presented as mean ± standard deviation. Significant differences (Student’s t-test, p<0.05).

^1^ Derived from linear regression adjusted for baseline value at the age of 1 month (if applicable) and gender, 95% confidence interval in parentheses.

At 4 years of age, there were no significant differences in anthropometry and body composition parameters between groups in intention-to-treat (Table 2) and per-protocol population (S2 Table). Adjustment for gender and confounders did not change this finding.

BMI was positively related to the percentage of body fat estimated by BIA or skinfolds, while there was a stronger correlation for skinfolds (R = 0.62, p<0.001) than for BIA (R = 0.36, p<0.001). Body fat percentage estimates obtained by both methods correlated well (R = 0.50, p<0.001).

**Table 2:** Anthropometry and body composition at 4 years of age (intention-to-treat population).

	Formula group								Estimated difference between IF and CF ^2^	Breastfed group			
	Intervention				Control								
	n	Mean ± SD			n	Mean ± SD				n	Mean ± SD		
**Weight**													
(kg)	65	17.7	±	2.45	59	17.9	±	2.31	-0.15 (-0.96,0.66);p = 0.72	63	17.9	±	2.29
(z-score)	65	0.57	±	1.00	59	0.64	±	0.94	-0.08 (-0.41,0.25);p = 0.65	63	0.65	±	0.91
**Height**													
(cm)	65	108	±	4.45	59	108	±	4.45	-0.31 (-1.76,1.13);p = 0.67	63	108	±	1.05
(z-score)	65	1.11	±	1.05	59	1.25	±	1.05	-0.07 (-0.42,0.27);p = 0.67	63	1.05	±	0.94
**Head**													
(cm)	65	51.2	±	1.61	59	51.2	±	1.33	0.03 (-0.41,0.47);p = 0.89	63	51.3	±	1.22
**BMI**													
(kg/m^2^)	65	15.2	±	1.66	59	15.2	±	1.22	0.07 (-0.46,0.60);p = 0.80	63	15.4	±	1.56
(z-score)	65	-0.14	±	1.15	59	-0.16	±	0.91	-0.04 (-0.40,0.33);p = 0.85	63	0.02	±	1.12
**Weight-for-height**													
(z-score)	65	-0.16	±	1.12	59	-0.19	±	0.87	-0.03 (-0.39,0.33);p = 0.86	63	0.01	±	1.08
**BIA measurement**													
Body fat (kg)	65	2.21	±	1.33	59	2.07	±	1.12	0.15 (-0.29,0.58);p = 0.51	62	2.26	±	1.33
Lean mass (kg)	65	15.5	±	2.19	59	15.8	±	2.05	-0.23 (-0.99,0.54);p = 0.56	62	15.7	±	1.82
Body fat from BIA (%)	65	12.3	±	6.52	59	11.5	±	5.56	0.84 (-1.31,2.98);p = 0.44	62	12.3	±	5.77
**Skinfolds**													
Triceps (mm)	64	9.51	±	2.58	59	9.14	±	2.25	0.35 (-0.48,1.18);p = 0.41	63	9.48	±	2.46
Subscapular (mm)	64	6.16	±	2.12	59	6.01	±	1.75	0.15 (-0.54,0.83);p = 0.67	63	6.26	±	2.19
Body fat from skinfolds (%) ^1^	64	15.0	±	3.73	59	14.6	±	3.19	0.43 (-0.76,1.63);p = 0.48	63	15.1	±	3.64

IF, intervention formula; CF, control formula; BIA, bioelectrical impedance analysis; BMI, body mass index. Data presented as mean ± standard deviation. Significant differences (Student’s t-test, p<0.05)

^1^ Slaughter et al. 1988 [13].

^2^ Derived from linear regression adjusted for baseline value at the age of 1 month (if applicable), gender, age at visit, maternal age and maternal smoking status, 95% confidence interval in parentheses.

### Serious illnesses

The incidence of serious illnesses (any hospitalisation up to 4 years of age) was comparable between all groups (p = 0.25). No serious illness was observed in the IF group, while 3 children in the CF group (two children with infections, one child with mental disorder) and 2 children in the BF group (one child with infection, one child with hypothyroidism) developed one serious illness. Additionally, one child in the CF group developed two serious illnesses (cryptorchidism and arteria pulmonalis stenosis). We found no indications for a causal role of the study formula being responsible for any of these adverse events.

### Plasma acyl-carnitines and branched-chain amino acids

A total of 250 plasma samples of infants aged 4 months (IF: 80, CF: 79, BF: 91) were analyzed to investigate 59 different carnitine esters and 425 phospholipids. Fourteen carnitine esters had intra-batch and inter-batch coefficients variation of less than 30% and thus were considered for further statistical analysis. Absolute concentrations of phospholipids, sphingomyelins [14] and of plasma amino acids [11] have been published previously.

Four acyl-carnitines showed statistically significant group differences (Table 3). Acyl-carnitine C8:1, C12:0 and C14:0 were significantly higher in the IF group compared to the CF group, while acyl-carnitine C3:0 was significantly lower in the IF compared to CF infants.

**Table 3:** Median (interquartile range, IQR) carnitine and carnitine ester concentrations (μmol/L) of infants in the intervention, control and breastfed group at the age of 4 months of the intention-to-treat population.

		Formula Group							p (Intervention vs. Control)	Breastfed			
	Intervention				Control								
	n	Median	IQR		n	Median	IQR			n	Median	IQR	
**Free carnitine**	80	48.9	42.8	53.3	79	50.7	42.0	55.8	0.41	91	44.0	40.2	50.1
**Total acyl-carnitines**	80	10.3	9.52	11.6	79	10.2	8.77	11.1	0.11	91	10.2	8.67	11.5
**Short-chain acyl-carnitines (<C5)**	80	9.04	8.11	10.2	79	8.93	7.86	9.78	0.35	91	9.18	7.69	10.2
Carn.a.C2.0	80	8.04	7.20	9.42	79	7.83	6.65	8.59	0.13	91	8.47	6.98	9.36
Carn.a.C3.0	80	0.68	0.59	0.80	79	0.79	0.69	0.90	**0.0002**	91	0.62	0.47	0.74
Carn.a.C4.0	80	0.25	0.20	0.28	79	0.25	0.22	0.32	0.42	91	0.19	0.14	0.24
Carn.a.C8.1	80	0.73	0.41	0.91	79	0.42	0.31	0.52	**<0.0001**	91	0.14	0.09	0.23
**Long-chain acyl-carnitines (≥C10)**	80	0.78	0.63	0.98	79	0.77	0.68	0.93	0.89	91	0.78	0.69	0.99
Carn.a.C10.1	80	0.21	0.16	0.27	79	0.24	0.19	0.31	0.01	91	0.22	0.17	0.31
Carn.a.C12.0	80	0.11	0.08	0.16	79	0.09	0.06	0.11	**0.0002**	91	0.10	0.08	0.16
Carn.a.C14.0	80	0.03	0.02	0.04	79	0.02	0.01	0.03	**0.0004**	91	0.04	0.03	0.05
Carn.a.C14.1	80	0.12	0.10	0.14	79	0.12	0.10	0.15	0.85	91	0.12	0.10	0.14
Carn.a.C14.2	80	0.03	0.02	0.04	79	0.03	0.02	0.04	0.48	91	0.04	0.03	0.05
Carn.a.C16.0	80	0.10	0.08	0.12	79	0.09	0.08	0.10	0.05	91	0.09	0.07	0.11
Carn.a.C16.1	80	0.02	0.01	0.03	79	0.02	0.01	0.02	0.47	91	0.02	0.02	0.03
Carn.a.C18.0	80	0.03	0.03	0.04	79	0.03	0.02	0.04	0.15	91	0.04	0.03	0.05
Carn.a.C18.1	80	0.12	0.09	0.14	79	0.11	0.10	0.13	0.85	91	0.10	0.08	0.12
Carn.a.C2.0/total acyl-carnitines	80	0.76	0.75	0.79	79	0.77	0.75	0.78	0.58	91	0.82	0.79	0.84
Long-chain acyl-carnitines/total acyl-carnitine	80	0.08	0.06	0.09	79	0.08	0.07	0.09	0.38	91	0.08	0.07	0.10
Carn.a.C2.0/free carnitine	80	0.16	0.14	0.19	79	0.15	0.13	0.19	0.05	91	0.18	0.16	0.22
Long-chain acyl-carnitines/free carnitine	80	0.02	0.01	0.02	79	0.02	0.01	0.02	0.36	91	0.02	0.01	0.02

Data are presented as medians and interquartile ranges. P-values were computed with the use of the Kruskal-Wallis rank test (Bonferroni-corrected p-value of p<0.00038). Acyl-carnitines with a chain length ≥C10 are defined as long-chain acyl-carnitines, and with a chain length <C5 as short-chain acyl-carnitines.

### Metabolites in relation to growth

To identify predictors of later anthropometry measures, associations between metabolites and absolute anthropometric outcome at 4 months or 4 years of age or between metabolites and growth (change in z-score between 1 month and 4 months or 4 months and 4 years) were performed using linear regression and Bonferroni-corrected p-values of p<0.00038.

The analyses on the infant metabolome at the age of 4 months and prediction of anthropometry up to 4 years of age showed no association between metabolites and long-term growth (absolute anthropometric parameter at 4 years of age and change in growth between 4 months and 4 years of age).

Short-term growth up to 4 months of age indicated no association between metabolites and absolute anthropometric outcome at 4 months of age. Furthermore, no correlation was found between metabolites and the change in length-for-age or weight-for-length z-scores between 1 month and 4 months of age. However, a correlation was found between metabolites and change in weight-for-age and BMI-for-age z-score during the same time period (Table 4) A total of 12 metabolites (8 amino acids, free carnitine, 3 phosphatidylcholines) showed significant correlation with change in weight-for-age and BMI-for-age z-score from 1 to 4 months of age (Table 4). Including feeding group as adjusted variable, 6 metabolites remained significantly associated with the change in weight-for-age z-score (Asn, Lys, Met, Phe, Trp, Tyr) and BMI-for-age z-score (Tyr only).

**Table 4:** Associations between metabolites and change in weight and BMI z-score from 1 month to 4 months with and without adjustment for feeding group.

	Without adjustment for feeding group			With adjustment for feeding group		
	Regression coefficient	(95% CI)	p-value	Regression coefficient	(95% CI)	p-value
*Metabolites related to change in weight*						
PCaec38:4	-0.044	(-0.065,-0.023)	0.0001	-0.045	(-0.071,-0.018)	0.001
PCaec40:4	-0.327	(-0.502,-0.152)	0.0003	-0.303	(-0.512,-0.094)	0.005
PCaec40:5	-0.196	(-0.293,-0.099)	0.0001	-0.193	(-0.312,-0.074)	0.002
Free carnitine	0.021	(0.010,0.031)	0.0002	0.019	(0.008,0.029)	0.0009
Asn	0.017	(0.010,0.024)	<0.0001	0.016	(0.008,0.024)	0.0001
Ile	0.009	(0.004,0.013)	0.0001	0.008	(0.004,0.013)	0.0005
Leu	0.005	(0.002,0.007)	0.0001	0.004	(0.002,0.007)	0.0007
Lys	0.004	(0.003,0.006)	<0.0001	0.004	(0.002,0.006)	<0.0001
Met	0.023	(0.012,0.033)	<0.0001	0.022	(0.010,0.033)	0.0002
Phe	0.015	(0.009,0.021)	<0.0001	0.014	(0.008,0.020)	<0.0001
Trp	0.017	(0.010,0.024)	<0.0001	0.017	(0.010,0.024)	<0.0001
Tyr	0.010	(0.007,0.014)	<0.0001	0.010	(0.006,0.014)	<0.0001
*Metabolites related to change in BMI*						
Lys	0.005	(0.002,0.007)	0.0003	0.005	(0.002,0.007)	0.0006
Phe	0.015	(0.007,0.023)	0.0003	0.014	(0.006,0.023)	0.0009
Tyr	0.010	(0.005,0.015)	0.0002	0.010	(0.005,0.015)	0.0003

Only metabolites significantly related to weight-for-age/BMI-for-age z-score change resulting from the unadjusted linear regression model are listed.

Only the association between change in weight between 1 and 4 months of age and Trp or Tyr indicating a significant p-value met the Bonferroni-corrected p-value threshold when additional adjustments for baseline weight-for-age z-score/BMI-for-age z-score and smoking status during pregnancy were done in metabolites identified in Table 4.

## Discussion

The short-term evaluation of an intervention formula with a lower protein content, containing alpha-lactalbumin enriched whey and LC-PUFA, showed differential growth effects compared to a normal protein control formula up to the age of 4 months [11]. In contrast, no group difference was detected at the age of 4 years.

### Anthropometry at 4 years and comparison to other studies

A high rate of 72.2% of children from the main study participated in the FU study with a significantly higher percentage of children originating from the per-protocol than the intention-to-treat population (p = 0.02). The obesity rate of 6.95% is comparable to that reported in another Serbian study [16]. The identified correlation between BIA and skinfold measurement was similar to Orta et al. (2014) in children aged 9.5 years (R = 0.74, p<0.001) [17]. The current evidence concerning the effect of protein intake during infancy on long-term body composition outcome is not clear [18].

Children previously fed IF showed adequate growth up to 4 years of age that was not different from growth compared to the other study group. While the average growth rate (change in weight-for-age, length-for-age z-score) during the first 4 months (intervention period) was higher in IF compared to CF infants (discussion see [11]), the growth of IF children slowed down compared to CF children between 4 months and 4 years of age (FU period) suggesting a catch-down growth effect. Furthermore, change in BMI did not differ during the intervention and FU period indicating a proportionally higher growth of weight and length in IF infants. Interestingly, Putet et al. [19] compared infants fed with a standard formula (2.7 g protein/100 kcal) to those receiving a low-protein formula (1.8 g protein/100 kcal) and found no impact on growth velocities during the first year of life, anthropometry at 4 years of age (expressed as z-scores) and body composition at 3 or 5 years of age [19]. Also the IGF-I concentrations (marker linking effects of nutrition to growth in infants) at 4 and 9 months of age, did not differ between the formula groups [19]. Putet et al. speculated that the difference in protein contents of study formulae may have been too small to find a difference in plasma IGF-I concentration and related growth [19].

### Acyl-carnitines as degradation products of branched-chain amino acids

Group differences in acyl-carnitine concentrations in our study may be explained by a compositional difference in the study formulae (S3 Table). The higher plasma content of acyl-carnitine C3.0 in infants receiving the standard formula may be a result of the higher amino acid content in this formula. Furthermore, the higher contents of acyl-carnitine C8.1, C12.0 and 14.0 in infants receiving the IF might be related to its higher content of medium-chain fatty acids. Similar observations were reported in the study by Bene et al., in which plasma concentrations of medium-chain acyl-carnitines were related to the formula content of medium-chain triglycerides [20].

Branched-chain amino acids are preferentially oxidized in muscle [21]. In infants aged 6 months, the catabolism of branched-chain amino acids to its respective degradation products follows saturation kinetics, which at very high plasma branched-chain amino acid concentration might result in adverse regulatory effects [9]. Inhibiting effects on the beta-oxidation of fatty acids might increase body fat storage [9]. However, the amino acid and carnitine ester concentrations found in the BeMIM infants did not show indications of saturation kinetics. Only four infants had Ile concentrations above the previously reported breakpoint concentration of 136 μmol/L, and only two infants had Leu concentrations above the previously reported breakpoint concentration of 234 μmol/L [9]. This is likely due to the fact that the protein content of the BeMIM control formula was lower (2.2 g/100 kcal) than the high protein formula studied by Kirchberg et al. [9] (infant formula 2.9 g/100 kcal, follow-on formula 4.4 g/100 kcal).

### Metabolic parameters associated with early growth

In the BeMIM study, plasma metabolite concentrations at 4 months of age did not show any relation to anthropometry at 4 months or 4 years of age. No long-term effects of measured metabolite concentrations on anthropometry at 4 years of age were found. This is in contrast to Rzehak et al. [10], who identified lysophosphatidylcholine LPCaC14:0 at 6 months of age as being a significantly related risk of overweight or obesity at 6 years of age.

In respect to short-term effects of metabolite concentrations on anthropometry, Rzehak et al. 2014 [10] reported relations between 19 different metabolites at 6 months of age and change in weight-for-age z-score from birth to 6 months of age. We tried to replicate these findings in the BeMIM infants that differed only slightly in age (BeMIM cohort was 2 months younger).

In the BeMIM study, a total of 12 metabolites (8 amino acids, free carnitine, 3 phosphatidylcholines) were identified to show significant associations between infant weight or BMI z-score change from 1 to 4 months of age. These associations might primarily be attributed to the difference between breastfed and formula-fed infants.

The association of 8 plasma amino acids (Asn, Ile, Leu, Lys, Met, Phe, Trp, Tyr) and their relationship to growth might be explained by the higher protein intake and higher growth rates of formula-fed infants compared to breastfed infants. Data from previous studies indicate that a higher amino acid and energy intake in formula-fed infants is associated with higher infant growth velocities [7] [22]. Especially branched-chain amino acids, aromatic amino acids, and also Lys are thought to contribute to the change in weight and BMI via stimulating the secretion of insulin [14,23,24].

Plasma carnitine levels in study subjects were within published reference values (31 to 61 nmol/mL) [25]. Plasma carnitine derives from exogenous sources like human milk or cow’s milk-based infant formula, or can be synthesized endogenously from the amino acid precursors Lys and Met [25]. The carnitine supply from cow’s milk-based infant formula is comparable or slightly higher compared to that of human milk [26]. High bioavailability of dietary carnitine in infant diets and a balanced protein supply are important to ensure adequate carnitine status, as the endogenous production of carnitine alone is considered insufficient to support stable plasma free carnitine concentration in infancy [25].

Rzehak et al. 2014 reported that phosphatidylcholines PCaec38:4, Pcaec40:4, Pcaec40:5 are inversely related to weight change up to 6 months of age [10], but did not identify a potential underlying mechanism.

In the BeMIM cohort, Asn, Lys, Met, Phe, Trp and Tyr were significantly associated with change in weight-for-age z-score between 1 and 4 months of age after adjustment for feeding group. Of these amino acids, only Tyr was associated with change in BMI-for-age z-score. The change in BMI-for-age z-score might be an indicator of later obesity [6]. In obese children, plasma Tyr concentrations are positively associated with homeostasis model assessment (HOMA), which might be explained by the effect of insulin on Tyr amino transferase activity [27]. Elevated Tyr levels might be used as an early biomarker for metabolic syndrome in young adults [28], while long-term effects of Tyr on insulin resistance and related metabolism and obesity require further clarification.

### Limitations

One limitation of the BeMIM FU was that the sample size was calculated to detect a difference in growth during the first 4 months (main study), but not for the FU outcomes measured at 4 years of age. However, the follow-up rate between 4 months and 4 years of age was about 72.3% (IF 76.5%, CF 72.0, BF 68.5%). Furthermore, recent findings indicate that the FU time point at 4 years of age might be in a phase of slow growth followed by an adiposity rebound [29]. Consequently, findings might differ when looking at growth during an even later period.

### Conclusions

In contrast to previous evidence indicating that early protein intake and weight gain have an impact on later obesity risk [22], results of the current study do not indicate long-term effects of early diet and growth on anthropometry at 4 years of age. The 4-year FU of the BeMIM study showed that the administration of a new formula with a lower protein content, a higher content of alpha-lactalbumin enriched whey and containing LC-PUFA does not produce measurable growth differences in children compared with children receiving a standard protein content formula during the first 4 months of life.

### Ethical standards

Manuscripts submitted for publication include data of the BeMIM study (Fleddermann et al. 2014, [11]). The study was conducted according to the guidelines laid down in the Declaration of Helsinki 1964 and all procedures involving human subjects were approved by the ethical committee of the Clinical Center Serbia Ethical Committee. Written informed consent was obtained from all parents prior to study start after the experimental protocol had been explained to them in detail.

## Supporting information

S1 Checklist CONSORT 2010 checklist. (PDF)

S1 Table Baseline characteristics (intention-to-treat population) of follow-up infants. (DOCX)

S2 Table Anthropometry and body composition at 4 years of age (per-protocol population). (DOCX)

S3 Table Amino acid, fatty acid and micronutrient composition of study formulae. (DOCX)

S1 Protocol Study protocol plus amendments. (PDF)

## Abbreviations

BIA: bioelectrical impedance analysis
BMI: body mass index
CF: control formula
FU: follow-up
IF: intervention formula
LC-PUFA: long-chain polyunsaturated fatty acids
