# Supplementary material for: Association of infant formula composition and anthropometry at 4 years: Follow-up of a randomized controlled trial (BeMIM study)
Source: PLoS One. 2018 Jul 5;13(7):e0199859. doi: 10.1371/journal.pone.0199859 (PMC6033437; doi:10.1371/journal.pone.0199859)
Supplement: S1 Table — (DOCX) [file pone.0199859.s002.docx]

**S1 Table** Baseline characteristics (intention-to-treat population) of follow-up infants.

|  | **Formula group** | | | | | | | | | | |  |  |  | **Breastfed group** | | | | |  |
| --- | --- | --- | --- | --- | --- | --- | --- | --- | --- | --- | --- | --- | --- | --- | --- | --- | --- | --- | --- | --- |
|  | **Intervention** | | | | |  | **Control** | | | | |  | **p value** |  |  |  |  |  |  |  |
|  | n | (%) | Mean ± SD | | |  | n | (%) | Mean ± SD | | |  | IF vs. CF |  | n | (%) | Mean ± SD | | |  |
| **Total^1^** | 65 | (79.3) | ― | | |  | 59 | (72.0) |  | ― |  |  | 0.27 |  | 63 | (68.5) |  | ― |  |  |
| **Age** (y) | 65 |  | 3.95 | ± | 0.03 |  | 59 |  | 3.95 | ± | 0.13 |  | 0.36 |  | 63 |  | 3.97 | ± | 0.03 |  |
| **Male sex** | 34 | (52.3) | ― | | |  | 30 | (50.8) |  | ― |  |  | 0.87 |  | 32 | (50.8) |  | ― |  |  |
| **Maternal education** |  |  |  |  |  |  |  |  |  |  |  |  |  |  |  |  |  |  |  |  |
| Basic/Additional/Tertiary | 4/40/21 | | ― | | |  | 2/37/20 | |  | ― |  |  | 0.77 |  | 0/24/38 | |  | ― |  |  |
| **Mother smoked** |  |  |  |  |  |  |  |  |  |  |  |  |  |  |  |  |  |  |  |  |
| Yes | 24 | (36.9) | ― | | |  | 23 | (39.0) |  | ― |  |  | 0.81 |  | 19 | (30.2) |  | ― |  |  |
| if Yes, duration (y) | 24 |  | 12.0 | ± | 7.0 |  | 22 |  | 12.8 | ± | 5.2 |  | 0.69 |  | 19 |  | 13.1 | ± | 5.4 |  |
| **Age mother** (y) | 65 |  | 31.1 | ± | 5.1 |  | 59 |  | 31.9 | ± | 5.1 |  | 0.40 |  | 63 |  | 31.7 | ± | 4.5 |  |
| **BMI mother** (kg/m²) | 61 |  | 23.4 | ± | 3.6 |  | 59 |  | 23.2 | ± | 3.9 |  | 0.71 |  | 62 |  | 23.1 | ± | 3.9 |  |
| **Father smoked** |  |  |  |  |  |  |  |  |  |  |  |  |  |  |  |  |  |  |  |  |
| Yes | 20 | (30.8) | ― | | |  | 25 | (42.4) |  | ― |  |  | 0.18 |  | 23 | (36.5) |  | ― |  |  |
| if Yes, duration (y) | 20 |  | 15.8 | ± | 7.9 |  | 25 |  | 16.0 | ± | 6.3 |  | 0.96 |  | 23 |  | 18.6 | ± | 7.5 |  |
| **Age father** (y) | 59 |  | 34.5 | ± | 5.9 |  | 58 |  | 34.4 | ± | 5.6 |  | 0.92 |  | 62 |  | 34.6 | ± | 6.2 |  |
| **BMI father** (kg/m²) | 57 |  | 27.0 | ± | 4.7 |  | 55 |  | 26.7 | ± | 3.3 |  | 0.75 |  | 61 |  | 27.6 | ± | 4.7 |  |
|  |  |  |  | | |  |  |  |  |  |  |  |  |  |  |  |  |  |  |  |

IF, intervention formula; CF, Control formula; BMI, body mass index. Data are presented as mean ± standard deviation. Significant difference between formula and breastfed group (p<0.05). P values were computed with the use of Student´s t-test. Chi-square test was used for categorical data. ^1^of infants finished study at 4 months.
