# Supplementary material for: Association of infant formula composition and anthropometry at 4 years: Follow-up of a randomized controlled trial (BeMIM study)
Source: PLoS One. 2018 Jul 5;13(7):e0199859. doi: 10.1371/journal.pone.0199859 (PMC6033437; doi:10.1371/journal.pone.0199859)
Supplement: S2 Table — (DOCX) [file pone.0199859.s003.docx]

**S2 Table** Anthropometry and body composition at 4 years of age (per-protocol population).

|  | **Formula group** | | | | | | | | |  | **p value**  **(t test)** | **Estimated difference between IF and CF ^2^** |  | **Breastfed group** | | | |  |
| --- | --- | --- | --- | --- | --- | --- | --- | --- | --- | --- | --- | --- | --- | --- | --- | --- | --- | --- |
|  | **Intervention** | | | |  | **Control** | | | |  |  |  |  |  |  |  |  |  |
|  | n | Mean ± SD | | |  | N | Mean ± SD | | |  | IF vs. CF |  |  | n | Mean ± SD | | |  |
|  |  |  |  |  |  |  |  |  |  |  |  |  |  |  |  |  |  |  |
| **Weight** |  |  |  |  |  |  |  |  |  |  |  |  |  |  |  |  |  |  |
| (kg) | 42 | 17.9 | ± | 2.72 |  | 25 | 17.6 | ± | 2.64 |  | 0.60 | 0.27 (-1.10,1.65);p=0.69 |  | 40 | 18.0 | ± | 2.14 |  |
| (z-score) | 42 | 0.62 | ± | 1.10 |  | 25 | 0.50 | ± | 1.08 |  | 0.66 | 0.10 (-0.46,0.66); p=0.71 |  | 40 | 0.70 | ± | 0.83 |  |
| **Height** |  |  |  |  |  |  |  |  |  |  |  |  |  |  |  |  |  |  |
| (cm) | 42 | 108 | ± | 4.60 |  | 25 | 108 | ± | 5.27 |  | 0.92 | -0.13 (-2.44,2.19);p=0.91 |  | 40 | 108 | ± | 3.93 |  |
| (z-score) | 42 | 1.17 | ± | 1.10 |  | 25 | 1.22 | ± | 1.24 |  | 0.88 | -0.02 (-0.57,0.53);p=0.94 |  | 40 | 1.07 | ± | 0.93 |  |
| **Head** |  |  |  |  |  |  |  |  |  |  |  |  |  |  |  |  |  |  |
| (cm) | 42 | 51.3 | ± | 1.55 |  | 25 | 51.0 | ± | 1.64 |  | 0.45 | 0.08 (-0.63,0.78);p=0.83 |  | 40 | 51.5 | ± | 1.28 |  |
| **BMI** |  |  |  |  |  |  |  |  |  |  |  |  |  |  |  |  |  |  |
| (kg/m²) | 42 | 15.3 | ± | 1.81 |  | 25 | 14.9 | ± | 1.27 |  | 0.38 | 0.24 (-0.59,1.08);p=0.56 |  | 40 | 15.5 | ± | 1.60 |  |
| (z-score) | 42 | -0.12 | ± | 1.23 |  | 25 | -0.34 | ± | 0.98 |  | 0.47 | 0.14 (-0.45,0.72);p=0.64 |  | 40 | 0.07 | ± | 1.14 |  |
| **Weight-for-height** |  |  |  |  |  |  |  |  |  |  |  |  |  |  |  |  |  |  |
| (z-score) | 42 | -0.14 | ± | 1.20 |  | 25 | -0.38 | ± | 0.94 |  | 0.41 | 0.19 (-0.36;0.75);p=0.49 |  | 40 | 0.06 | ± | 1.10 |  |
| **BIA measurement** |  |  |  |  |  |  |  |  |  |  |  |  |  |  |  |  |  |  |
| Body fat (kg) | 42 | 2.09 | ± | 1.37 |  | 25 | 2.15 | ± | 1.17 |  | 0.85 | 0.08 (-0.59,0.74);p=0.82 |  | 40 | 2.24 | ± | 1.37 |  |
| Lean mass (kg) | 42 | 15.8 | ± | 2.27 |  | 25 | 15.4 | ± | 2.14 |  | 0.45 | 0.51 (-0.66,1.68);p=0.39 |  | 40 | 15.8 | ± | 1.66 |  |
| Resistance (ohm) | 42 | 723 | ± | 81.2 |  | 25 | 751 | ± | 87.4 |  | 0.20 | -25.7 (-69.3,17.8);p=0.24 |  | 40 | 724 | ± | 77.0 |  |
| Basal metabolic rate (kcal) | 42 | 916 | ± | 107 |  | 25 | 947 | ± | 103 |  | 0.25 | 7.80 (-17.9,33.5);p=0.55 |  | 40 | 929 | ± | 93.2 |  |
| Body fat from BIA (%) | 42 | 11.4 | ± | 6.13 |  | 25 | 12.0 | ± | 5.62 |  | 0.69 | -0.03 (-3.07,3.02);p=0.99 |  | 40 | 12.1 | ± | 6.11 |  |
| **Skinfolds** |  |  |  |  |  |  |  |  |  |  |  |  |  |  |  |  |  |  |
| Triceps (mm) | 41 | 9.32 | ± | 2.68 |  | 25 | 9.18 | ± | 1.95 |  | 0.82 | 0.47 (-0.73,1.67);p=0.44 |  | 40 | 9.35 | ± | 2.27 |  |
| Subscapular (mm) | 41 | 6.07 | ± | 2.47 |  | 25 | 6.11 | ± | 2.20 |  | 0.96 | 0.22 (-0.99,1.42);p=0.72 |  | 40 | 6.34 | ± | 2.35 |  |
| Body fat from skinfolds (%) ^1^ | 41 | 14.7 | ± | 4.04 |  | 25 | 14.7 | ± | 3.16 |  | 0.95 | 0.53 (-1.34,2.40);p=0.57 |  | 40 | 15.0 | ± | 3.71 |  |

IF, intervention formula; CF, Control formula; BIA, bioelectrical impedance analysis; BMI, body mass index. Mean ± standard deviation. Significant differences (Student´s t-test, p<0.05) ^1^ Slaughter et al. 1988. ^2^ Derived from linear regression adjusted for baseline value at the age of 1 month (if applicable), gender, age at visit, maternal age and maternal smoking status, 95% confidence interval in parentheses.
