# Supplementary material for: Association of infant formula composition and anthropometry at 4 years: Follow-up of a randomized controlled trial (BeMIM study)
Source: PLoS One. 2018 Jul 5;13(7):e0199859. doi: 10.1371/journal.pone.0199859 (PMC6033437; doi:10.1371/journal.pone.0199859)
Supplement: S3 Table — (DOCX) [file pone.0199859.s004.docx]

**S3 Table** Amino acid, fatty acid and micronutrient composition of study formulae.

|  |  | **Intervention** | **Control** |
| --- | --- | --- | --- |
| Energy | kcal/100mL | 67 | 67 |
| Whey:casein ratio |  | 60:40 | 60:40 |
| Protein | g/100mL | 1.3 | 1.5 |
| Protein | g/100kcal | 1.89 | 2.2 |
| Carbohydrate | g/100mL | 7.5 | 7.8 |
| Fat | g/100mL | 3.6 | 3.3 |
| Sodium | mg/100mL | 21 | 25 |
| Potassium | mg/100mL | 69 | 77 |
| Calcium | mg/100mL | 52 | 62 |
| Phosphorus | mg/100mL | 35 | 41 |
| Calcium:Phosphorus | - | 1.5 | 1.5 |
| Chloride | mg/100mL | 46 | 48 |
| Magnesium | mg/100mL | 5.6 | 6.1 |
| Zinc | mg/100mL | 0.6 | 0.6 |
| Ferric | mg/100mL | 0.6 | 0.8 |
| Copper | µg/ 100mL | 36 | 26 |
| Iodine | µg/ 100mL | 13 | 12 |
| Manganese | µg/ 100mL | 8.6 | 1.6 |
| Selenium | µg/ 100mL | 2.2 | 1.0 |
| Fluoride | µg/ 100mL | 6.9 | 3.5 |
| **Amino acids** |  |  |  |
| Alanine | mg/100mL | 52 | 66 |
| Arginine | mg/100mL | 37 | 43 |
| Aspartic acid | mg/100mL | 124 | 146 |
| Cysteine | mg/100mL | 24 | 27 |
| Glutamic acid | mg/100mL | 258 | 309 |
| Glycine | mg/100mL | 25 | 30 |
| Histidine | mg/100mL | 31 | 37 |
| Isoleucine | mg/100mL | 72 | 88 |
| Leucine | mg/100mL | 132 | 156 |
| Lysine | mg/100mL | 111 | 124 |
| Methionine | mg/100mL | 28 | 33 |
| Phenylalanine | mg/100mL | 73 | 63 |
| Proline | mg/100mL | 101 | 121 |
| Serine | mg/100mL | 68 | 82 |
| Threonine | mg/100mL | 69 | 88 |
| Tryptophan | mg/100mL | 25 | 24 |
| Tyrosine | mg/100mL | 48 | 51 |
| Valine | mg/100mL | 79 | 96 |
| **Fatty acids** |  |  |  |
| C12:0 | g/100 mL | 0.2 | 0 |
| C14:0 | g/100 mL | 0.1 | 0 |
| C16:0 | g/100 mL | 0.8 | 0.9 |
| C16:1 | g/100 mL | 0 | 0 |
| C18:0 | g/100 mL | 0.1 | 0.1 |
| C18:1 | g/100 mL | 1.5 | 1.4 |
| C18:2 | g/100 mL | 0.7 | 0.7 |
| C18:3 | g/100 mL | 0.1 | 0.1 |
| C20:4 | mg/100 mL | 7.2 | 0 |
| C22:6 | mg/100 mL | 7.2 | 0 |
