# Supplementary material for: Association of infant formula composition and anthropometry at 4 years: Follow-up of a randomized controlled trial (BeMIM study)
Source: PLoS One. 2018 Jul 5;13(7):e0199859. doi: 10.1371/journal.pone.0199859 (PMC6033437; doi:10.1371/journal.pone.0199859)
Supplement: S1 Protocol — (PDF) [file pone.0199859.s005.pdf]

BeMIM
Belgrade-Munich Infant Milk Trial

Narodni Front
Ginekološko akušerska klinika

HiPP

Study Protocol

Version 20.04.2009

Formula with modified content of protein and improved fatty acids and their impact on infant growth and health

Short Title of Study:
BeMIM – Belgrade-Munich Infant Milk Study

Study number:
502042

**Chief Investigator:**	Prof. Dr. Berthold Koletzko<br>LMU Munich University Hospital<br>Dr. von Hauner Children's Hospital<br>Munich, GERMANY.
**Principal Investigator:**	Dr. Nataša Raspopović<br>Ginekološko Akušerska Klinika "Narodni Front"<br>Hospital for Gynecology and Obstetrics<br>Belgrade, SERBIA.
**Study Sponsor:**	HiPP GmbH & Co. Vertrieb KG<br>Dr. Regina Berwind<br>Pfaffenhofen, GERMANY.
**Study Manager:**	Dr. Heike tom Dieck<br>HiPP GmbH & Co. Vertrieb KG<br>Pfaffenhofen, GERMANY.
**Project Coordinator:**	Dr. Branka Trišic<br>HiPP Representative Office<br>Bircaninova 17a, Belgrade, SERBIA.
**Monitor:**	Martina Scheer<br>LMU Munich University Hospital<br>Dr. von Hauner Children's Hospital<br>Munich, GERMANY.

Confidential
This protocol is intended for those specifically authorized to receive it. Any other use or disclosure is prohibited. All information is to be kept confidential.

Participating Institutions and Researchers

Study Coordination and biochemical analyses:

LMU Munich University Hospital
Dr. von Hauner Children's Hospital
Lindwurmstr. 4, 80337 Munich, GERMANY.

Prof. Dr. Berthold Koletzko (Chief Investigator) / Office
Tel.: +49 (0)89 5160 - 2826
Fax: +49 (0)89 5160 - 7742
E-Mail: office.koletzko@med.uni-muenchen.de

Martina Scheer (Monitor)
Tel.: +49 (0)89 5160 – 7760
Fax: +49 (0)89 5160 - 7742
E-Mail: Martina.Scheer@med.uni-muenchen.de

Dr. Hans Demmelmair (Laboratory Analysis)
Tel.: +49 (0)89 5160 - 3692
Fax: +49 (0)89 5160 - 3487
E-Mail: Hans.Demmelmair@med.uni-muenchen.de

Dr. Wolfgang Peissner (Laboratory Analysis)
Tel.: +49 (0)89 5160 - 3485
Fax: +49 (0)89 5160 - 3487
E-Mail: Wolfgang.Peissner@med.uni-muenchen.de

Dr. Veit Grote (Statistics)
Institut für Soziale Pädiatrie und Jugendmedizin
Heiglhofstr. 63
81377 München
Tel.: +49 (0)89 - 7100 - 9307
Fax: +49 (0)89 - 7100 - 9315
E-Mail: veit.grote@med.uni-muenchen.de

Dr. Astrid Rauh-Pfeiffer (Consultant)
Tel.: +49 (0)89 5160 - 7934
Fax: +49 (0)89 5160 - 4938
E-Mail: Astrid.Rau-Pfeiffer@med.uni-muenchen.de

Clinical Study Centre / Site:

Ginekološko Akušerska Klinika - “Narodni Front” Hospital for Gynaecology and Obstetrics
Kraljice Natalije 62, 11000 Belgrade, SERBIA.

Prof. Dušan Stanojević (Clinic Director)

Dr. Nataša Raspopović; Pediatrician (Principal Investigator)
Tel. Neonatol. Department: (00) 381 (011) 2068 - 228
Tel.: (00) 381 (0) 64 139 92 84
Fax: (00) 381 (0)11 2642 - 603
E-Mail: n.raspop@scnet.yu

Dr. Alexander Stanimirovic; Resident (Sub Investigator)
Tel.: (00) 381 (0)63 77 19 514
Fax: (00) 381 (0)11 2642 – 603
E-Mail: alesta115@hotmail.com

Study Sponsor:

HiPP GmbH & Co. Vertrieb KG
Georg-Hipp-Straße 7, 85276 Pfaffenhofen / Ilm, GERMANY.

Dr. Regina Berwind (Head of Nutrition Science Department)
Tel.: +49 (0)8441 757 - 303
Fax: +49 (0)8441 757 - 668
E-Mail: Regina.Berwind@hipp.de

Dr. Heike tom Dieck (Study Manager)
Tel.: +49 (0)8441 757 - 144
Fax: +49 (0)8441 757 - 777 – 144
Mobile: +49 (0)163 – 757 25 34
E-Mail Heike.tom-Dieck@hipp.de

Thea Weith (Assistant of Study Manager)
Tel.: +49 (0)8441 757 - 335
Fax: +49 (0)8441 757 - 777 - 335
E-Mail: Thea.Weith@hipp.de

Study Sponsor Representative in Serbia:

HiPP Representative Office
Bircaninova 17a, Belgrade, SERBIA.

Dr. Branka Triŝic (Local Project Coordinator)
Tel.: +381 (0)11 2642 - 603
Fax: +381 (0)11 2642 - 603
E-Mail: hipp@technicom.net

Investigator Agreement

I agree to conduct this clinical study in accordance with the design and specific provisions of this protocol; modifications to the study are acceptable only with a mutually agreed upon protocol amendment. I will provide copies of the protocol, any subsequent amendments and access to all information furnished by sponsor to study personnel under my supervision.

I will discuss this material with them to ensure that they are fully informed about the investigational study formula and the study protocol. I also agree to conduct this study in compliance with Good Clinical Practice (GCP) standards as defined by the International Conference on Harmonization (ICH) Guideline for Good Clinical Practice, all applicable national, state, and local regulations, as well as the requirements of the appropriate Institutional Review Board/Independent Ethics Committee (IRB/IEC) and any other institutional requirements.

I agree to await Ethic-Committee approval for the protocol before initiating the study, to obtain consent from subjects prior to their enrolment in the study, to collect and record data as required by this protocol and case report forms, to prepare adverse events reports as required and to maintain study documentation for the period of time required.

Place, Date of Signature (dd/mm/yy)	Dr. Nataša Raspopović<br>Principal Investigator

Study protocol approved:

Place, Date of Signature (dd/mm/yy)	Prof. Dr. Berthold Koletzko<br>Chief Investigator

TABLE OF CONTENT

PARTICIPATING INSTITUTIONS AND RESEARCHERS 2

INVESTIGATOR AGREEMENT 5

TABLE OF CONTENT 6

LIST OF ABBREVIATIONS 10

1 SUMMARY / SYNOPSIS 11

2 INTRODUCTION 15

3 STUDY OBJECTIVES 18

4 STUDY DESIGN 18

4.1 Type of study 18
4.2 Groups and subjects 18
4.3 Duration of subject participation 19

5 STUDY POPULATION 19

5.1 Description 19
5.2 Subject inclusion criteria 20
5.3 Subject exclusion criteria 20
5.4 Definition of Compliance 21
5.4.1 Definition of fully formula-feeding and fully breastfeeding 21
5.5 Definition of Non- Compliance 22
5.5.1 Severe non-Compliance (ITT Population) 22
5.5.2 Minor non-compliance (per protocol population) 22
5.5.3 Subject drop out 23

6 TREATMENT OF SUBJECTS 24

6.1 Product description 24
6.1.1 Composition 24
6.1.2 Form and dosage 25
6.1.3 Quality control and shelf life 26
6.1.4 Packaging and labelling 26
6.1.5 Blinding technique 26
6.2 Treatment administration 26
6.2.1 Amount, dose, frequency 26
6.2.2 Route of administration 27
6.2.3 Subject compliance 27
6.3 Concomitant diet and treatment 27
6.3.1 Concomitant diet 27
6.3.2 Concomitant treatments / medications 27
6.4 Product handling 28
6.4.1 Distribution and Storage 28
6.4.2 Product accountability and reconciliation 28

7 DEFINITION OF MEASURES ... 30

7.1 Primary Outcome ... 30

7.2 Secondary Outcomes ... 30
7.2.1 Anthropometric data ... 30
7.2.2 Protein status measurements ... 30
7.2.3 Fatty acid profile in plasma ... 30

7.3 Other measures ... 30
7.3.1 Physical Examination ... 30
7.3.2 Formula intake ... 31
7.3.3 Tolerance ... 31
7.3.4 Compliance to study protocol ... 32
7.3.5 Health observations ... 32
7.3.6 Formula associated observations ... 32

8 CONDUCT OF THE STUDY ... 34

8.1 Study plan, flow chart ... 34

8.2 Time schedule ... 34

8.3 Study initiation Visit ... 34

8.4 Parent information, Subject recruitment and Randomisation ... 34

8.5 Visit ... 36
8.5.1 Baseline Visit (4-5 days of life) ... 36
8.5.1.1 Inclusion and exclusion criteria ... 36
8.5.1.2 Core data ... 36
Feeding history from birth until recruitment ... 37
8.5.1.3 Anthropometric data ... 37
8.5.1.4 Physical examination ... 38
8.5.1.5 Blood withdrawal ... 38
8.5.1.6 Present feeding ... 39
8.5.1.7 Randomisation ... 39
8.5.1.8 Formula accountability ... 39
8.5.1.9 Reminder (Diary) ... 39
8.5.1.10 Reminder (Visit) ... 40
8.5.2 Visit 1 (Day 30±2), Visit 2 (Day 60±3) and Visit 3 (Day 90±3) ... 40
8.5.2.1 Health observations ... 40
8.5.2.2 Compliance to protocol ... 40
8.5.2.3 Formula associated observations ... 40
8.5.2.4 Adverse / Serious adverse events ... 41
8.5.2.5 Formula accountability ... 41
8.5.2.6 Reminder (Dairy) ... 41
8.5.2.7 Reminder (Visit) ... 41
8.5.3 Visit 4 (Day 120 ± 3) ... 41
8.5.3.1 Study termination ... 41

8.6 Diaries ... 42

8.7 Reminder call ... 42

8.8 Biological samples ... 43
8.8.1 Responsibilities, parameters and methods ... 43
8.8.2 Collection and preparation ... 44
8.8.2.1 Time of sample collection ... 44
8.8.2.2 Procedure of blood sampling ... 44
8.8.3 Logistics ... 45
8.8.4 Reporting of results ... 45

8.9 Breaking the code ... 46

8.10 Data management ... 46

8.10.1 Data on CRFs ... 46
8.10.2 Computerized data entry ... 46
8.10.3 Organization of data sheets ... 46
8.10.4 Unblinding procedure ... 47

9 STATISTICAL METHODS AND DATA ANALYSIS ... 48

9.1 Statistical background ... 48

9.2 Hypothesis to be tested ... 48

9.3 Primary outcome variable ... 48

9.4 Secondary outcome variables ... 48

9.5 Sample size calculation ... 48

9.6 Randomisation ... 49

9.7 Management of subject withdrawal ... 50

9.8 Data management ... 50

9.9 Statistical analysis ... 50

9.10 Methods secondary analysis ... 51

9.11 Complementary analysis ... 51

9.12 Responsibility of statistics ... 52

10 HANDLING OF ADVERSE EVENTS ... 53

10.1 Definition ... 53
10.1.1 Adverse event ... 53
10.1.2 Serious adverse event ... 53

10.2 Reporting and documentation ... 53
10.2.1 Reporting and documentation of adverse events (AE) ... 53
10.2.2 Reporting and documentation of serious adverse events (SAE) ... 55

11 LEGAL AND ETHICAL PRE-REQUIREMENTS ... 56

11.1 Legal requirements ... 56

11.2 Ethical aspects ... 56
11.2.1 Protection of subject's confidentiality ... 56

11.3 Informed consent ... 56

11.4 Ethics committee approval ... 57

11.5 Declaration of Helsinki ... 57

12 QUALITY ASSURANCE ... 57

12.1 GCP (Good clinical practice) ... 57

12.2 Internal Quality control ... 57

13 AGREEMENTS ... 58

13.1 Monitoring ... 58

13.2 Auditing ... 58

13.3 Study report and publications ... 59
13.3.1 Study report ... 59
13.3.2 Publications ... 59

13.4 Responsibilities 59
13.4.1 Overview 59
13.4.2 Responsibilities of the Principal Investigator 60
13.4.3 Responsibilities of the Sponsor 60
13.4.4 Responsibilities of the Chief Investigator (LMU) and team 61
13.4.5 Abnormal termination of the study 61

14 LITERATURE 62

15 APPENDICES 64

15.1 Appendix: Formula composition: Study Formula 64
15.2 Appendix: Formula composition: Control Formula 64
15.3 Appendix: Instruction for Preparation; Dosage and Drinking amounts 64
15.4 Appendix: Study Formula Inventory Form 64
15.5 Appendix: Flow Chart 64
15.6 Appendix: Time Event Schedule 64
15.7 Appendix: Parent Information 64
15.8 Appendix: Informed Consent 64
15.9 Appendix: Subject Screening List 64
15.10 Appendix: Subject Identification List 64
15.11 Appendix: Randomisation List 64
15.12 Appendix: Case report Form (CRF) 64
15.13 Appendix: Diaries Formula and breastfeeding group 64
15.14 Appendix: Protocol of the reminder call 64
15.15 Appendix: Amino acid profiling 64
15.16 Appendix: Fatty acid profiling 64
15.17 Appendix: Material provided for blood withdrawal 64
15.18 Appendix: SOP for Blood – Sample Collection and Handling 64
15.19 Appendix: Serious adverse events form 64
15.20 Appendix: Declaration of Helsinki 64
15.21 Appendix: Monitoring SOP 64
15.22 Appendix: Protocol Violation Form 64
15.23 Appendix: Study Formula Random Number List 64

LIST OF ABBREVIATIONS

AE	Adverse Event
BeMIM	Belgrade - Munich Infant Milk Study
CRF	Case Report Form
EC	Ethic Committee
GCP	Good Clinical Practice
IC	Informed Consent
ICH	International Conference on Harmonization
IEC	Independent Ethics Committee
IRB	Institutional Review Board
ISF	Investigator Site File
LCP	long chain polyunsaturated fatty acid
RCT	Randomised controlled trial
SAE	Serious Adverse Event
SOP	Standard Operating Procedure

1 Summary / Synopsis

Title of the clinical study Formula with modified content of protein and improved fatty acids and their impact of infant growth and health.

Study number 502042

Principal Investigator Dr. Nataša Raspopović
Ginekološko Akuŝerska Klinika - “Narodni Front” Hospital for Gynaecology and Obstetrics
Kraljice Natalije 62, 11000 Belgrade, SERBIA.

STUDY DESIGN

Scientific background Recent evidence suggests that higher protein intake during infancy might be associated with an increased risk of excessive weight gain later in life. In addition dietary intake of long-chain polyunsaturated fatty acids (LCP; ARA, C20:4n-6 and DHA, C22:6n-3) has been shown to be important for optimal infantile development.

It is aim to provide a formula with a composition more similar to that of breast milk. Therefore, in this study the suitability of an infant formula with modified content of protein and improved fatty acids for the particular nutritional use of healthy term infants will be investigated.

Study objectives Primary objective

…is to show non inferiority of an infant formula with a reduced protein content (1.89 g/100 kcal) compared to a standard infant formula with a protein content of 2.2 g/100 kcal referring to growth parameters of healthy term infants receiving this infant formulae fully in the first 4 month of life. As required by Art. 7 of the European Directive on Infant and Follow-on Formula 2006/141/EC for an Infant Formula with a protein content less than 2.0 g/100 kcal.

Secondary objectives

…is to show that parameters of protein status (serum

	albumine, blood urea nitrogen, serum creatinine, plasma amino acid-profiles) in blood of infants receiving the modified infant formula (protein: 1.89 g/100 kcal) instead of the standard formula (protein: 2.2 g/100 kcal) are closer to that of fully breastfed infants. Furthermore fatty acid-profile in plasma will be assessed and compared between the groups.
**Primary outcome**	Weight in grams will be assessed in all clinical study Visits. Average **weight gain / d** between Visit 1 (30 ± 2 days of life) and Visit 4 (120 ± 3 days of life) is the primary outcome.
**Secondary outcomes**	▪ Anthropometric data:<br>length (cm), head circumference (cm)<br>▪ Protein status measurements:<br>serum albumin, creatinine, blood urea nitrogen, plasma amino acid profile<br>▪ Fatty acid profile in plasma<br>▪ Formula intake, acceptance and tolerance<br>▪ Stool characteristics
**Study design**	The study is a double-blind, controlled, monocenter, prospective, randomised, parallel design, intervention study with two formula groups (modified formula = study group; standard formula = control group) and a reference group of breastfed infants.
**Number of subjects to be enrolled**	n = 100 / group = 300 subjects<br>100 breast fed infants, 100 infants receiving control formula and 100 infants receiving modified study formula
**Description of subjects**	Healthy, term newborns will be enrolled. Male and female subjects should be in all groups in equal portions.
**Inclusion criteria**	o Healthy term newborn<br>o gestational age between 37 and 41 weeks<br>o birth weight between the 3th and 97th weight-for-age

	percentile (≥ 3rd and ≤ 97th percentile) according to the EURO-Growth for boys and girls, respectively.<br>o Fully bottle-fed (at the latest with 28 days of age) and fully breast fed, respectively<br>o age of enrolment: 4th or 5th day of life<br>o written parental informed consent<br>o Serbian nationality
**Exclusion criteria**	o Malformations, congenital heart defect, congenital vascular disease, severe diseases of gastrointestinal tract, kidney, liver, central nervous system and / or metabolic disease<br>o Intensive care during first 14 days of life<br>o gestational age ≤ 37 and ≥ completed 41 weeks<br>o birth weight < 3rd and > 97th percentile<br>o participation in any other clinical study intervention<br>o twins, multiple birth<br>o neonatal infection<br>o medication and parenteral nutrition<br>o metabolic disorders<br>o birth-related complications<br>o severe disturbances of neonatal adaption
**Study flow**	Recruitment of non-breastfed children (potentially intended to skip breastfeeding or already formula-fed) until 5th day of life, and breastfed children included in the reference group. Children have to be in accordance with inclusion and exclusion criteria. Randomisation to one of the formula groups will be done according to a blinded randomisation plan. Intervention with formula for at least 3 month (until 120 days of age).<br>At 4-5 days of life (Baseline) and at 30, 60, 90 and 120 days of life, children will participate in a clinical study Visit. Medical examination will be performed and anthropometric data will be assessed. Blood samples will be collected at Baseline and Visit 4. During three days before each Visit, parents are asked

	to fill out a diary to assess amount of formula intake, formula acceptance and compliance to protocol. Between the Visits parents / caregivers will be called to assess formula intake, compliance to protocol and formula associated observations.
**Timetable**	Start of trial / first subject in: May 2009<br>End of trial / last subject in: September 2010
**<u>INTERVENTION</u>**	
**Intervention Formula**	Modified HiPP infant formula with a reduced protein content of 1.89 g/100 kcal and LCPs (arachidonic acid and docosahexaenoic acid, each 12.3 mg/100 kcal = 0.23% of fatty acids).<br>• In accordance with EU Directive 2006/141/EC on Infant Formulae and Follow-on Formulae and Codex Alimentarius standard for Infant Formula.
**Control Formula**	Control Formula: Standard HiPP infant formula with a protein content of 2.2 g/100 kcal, without LCPs. Commercial product.<br>• In accordance with Directive 1999/21/EC and in accordance with Codex Alimentarius standard for Infant Formula.
**Duration of intervention**	Formula will be given until 120 days of life (4 month)
**Amount, route of administration**	Formula will be given ad libitum and fully.

2 Introduction

Low protein in infant nutrition

An adequate supply of dietary protein is essential for healthy growth and development in infancy. Protein is the major functional and structural component of all cells, and amino acids play an important role as precursors of coenzymes, hormones, nucleic acids and other essential molecules[1].

Human milk is recognized as the optimal source of nutrients for infants throughout at least the first year of life[2]. The dietary reference intakes (DRI) for infants up to the age of 6 months are based upon data of breastfed infants receiving human milk as the principal source of nutrients[1].The protein DRI recommendation for infants is 1.52 g/kg/d, which is based upon an estimated average milk intake volume of 0.78 L/d and a mean protein content of human milk of 11.7 g/L during the first six month of life[1].

As amino acid composition of cow's milk protein, used in manufacturing infant formula, does not perfectly match the amino acid composition of human milk, infant formula contains more protein than breast milk. This is to guarantee that all amino acids supplied meet quantitatively the amounts of amino acid present in human milk.

The protein content reduction of the intervention formula has been made possible by the development of a protein mix based on cow's milk protein sources enabling to achieve an amino acid profile closer to that of human milk, in particular lowering threonine and increasing tryptophan levels. The highly nutritious native whey protein isolate has a high content of a-lactalbumin obtained by careful fractionation.

Recent evidence suggests that higher protein intakes during infancy might be associated with an increased risk of excessive weight gain. A higher habitual protein intake between the age of 12 and 24 months was associated with a higher body mass index (BMI) in girls, but not boys[3]. Furthermore, consistent high protein intake during the first year of life was associated with a larger BMI and increase in % body fat at the age of 7[4]. Breastfeeding has been associated with a modest, but protective effect against childhood obesity[5]. Speculations point towards the lower protein content of human milk as one possible protective reason.

The question of adequate protein intake in early life and obesity prevention has been addressed in the recent EU Childhood Obesity Project (CHOP) study[6]. In this multi-centre intervention study, involving five European countries with different habitual protein intakes more than 1000 infants were randomised either to a low protein formula (1.8 g/100 kcal and 2.2 g/100 kcal in the follow on formula) or a high protein formula (2.9 g/100 kcal and 4.4.g/100 kcal in the follow on formula). Additionally a non randomised reference group of breastfed infants was studied. Dietary intake and anthropometric development of the infants was followed until the age of 2 years. While energy intake was not different between the formula

groups, protein intake was significantly higher in the high protein formula group during the first 12 months of life. Length was not different between the randomised groups, but standard deviation scores of weight for length and body mass index were significantly higher in the high protein group than in the low protein group at age 2 years. During the first 2 years of life growth of infants receiving the low protein formula was more similar to growth of breast fed infants than was growth of high protein group infants.

Higher protein intakes throughout infancy may have a growth-stimulating effect[7]. It is being hypothesized that a protein intake in excess of metabolic needs might increase the secretion of insulin and insulin like growth factor 1 (IGF1), perhaps leading to enhanced growth during the first two years of life, which might predispose the growing infant to a higher risk of obesity in later life. Results of the CHOP study indicated that infants receiving high protein formulas showed significantly higher IGF-1 plasma concentrations and lower concentrations of insulin like growth factor binding protein 2 (IGF-BP2). The urinary excretion of C-peptide tended to be higher in infants with higher protein intakes at 3 months of age and was significantly higher at 6 months. The higher C-peptide concentration (relative to urinary creatinine concentration), indicates a greater degree of insulin secretion. Further studies are needed to clearly define the impact of high protein intake on infant metabolism.

In addition to the consequences of a higher protein intake on body weight and its implications on obesity in later life, excess protein intake in infancy may induce unnecessary stress on metabolism and renal function[i]. Several studies have shown that formula fed infants have higher urea nitrogen and plasma amino acid concentrations when compared to breast fed infants [8, 9, 10, 11, 12]. The negative consequences of a higher protein intake during infancy are reflected by the results of the recent CHOP study[6]. Three months old infants receiving high protein formulas showed significantly higher values for urinary osmolarity compared to their low-protein fed counterparts. Since the ability to concentrate urine is still limited in young infants, an increase in the urinary osmolarity should be avoided. As metabolic stress on the growing infant could be reduced, lower protein diets might be beneficial for the infants.

A number of clinical studies have investigated the impact of infant formulas with a lower protein concentration on outcome variables such as growth, tolerance and biochemical measures. Taken together, results from these studies indicate that healthy formula fed infants receiving a protein content of 1.8 g/100 kcal show no significant differences in weight, length, head circumference, serum albumin and nitrogen retention compared to infants receiving formulas containing 2.2g protein/100 kcal [12,13,,14,15]. On the other hand, a protein content of only 1.56 g/100 kcal during early infancy resulted in less gain in length compared to infants receiving a minimum level of 1.94 g/100 kcal[16]. These studies suggest that a protein concentration in infant formulas of at least 1.8 g/100 kcal results in similar growth pattern compared to an isocaloric higher protein formula[17]. However, the optimal protein content of infant formulas remains to be determined.

The lowest acceptable protein/energy ratio for cow`s milk-based infant formula, as defined by the EU Directive 2006/141/EG, is 1.8 g/100kcal (see below); the same value has been recommended by the European Society for Paediatric Gastroenterology, Hepatology[18] and Nutrition, the Codex Alimentarius[19] and the Committee on Nutrition of the American Academy of Pediatric[20]. However, according to the EU Directive, the suitability of an infant formula containing less than 2 g of protein per 100 kcal has to be shown in an appropriate research study.

Dietary intake of long-chain polyunsaturated fatty acids (LCP) such as arachidonic acid (ARA, C20:4n-6) and docosahexaenoic acid (DHA, C22:6n-3) has been shown to be important for optimal infantile development. If LCP intake through breastfeeding is not possible, because the infant is no longer breastfed, the use of an LCP-enriched formula is recommended by experts. The addition of LCPs enables formula-fed infants to reach an LCP status comparable with that of breastfed infants, which supports optimal visual and cognitive development[21]. The modified, low-protein infant formula will contain 12.3 mg/100 kcal LCPs, corresponding to 0.23% each ARA and DHA of total fatty acids. It is a secondary goal of the study, to investigate whether infants receiving the low-protein LCP enriched infant formula show higher LCP plasma phospholipids concentrations compared to infants receiving the standard formula without LCP and, thus, are more similar to fully breastfed infants.

In order to provide non breastfeed infants with the best nutrition possible the BEMIM trial is designed to 1) assess the suitability of a modified, low-protein infant formula (1.89 g/100 kcal) compared with a standard formula for the particular nutritional use of healthy term infants during the first four months of life and 2) to show that parameters of protein status are closer to that of breastfed infants.

3 Study Objectives

According to Article 7 of the European Directive on Infant and Follow-on Formula 2006/141/EC appropriate studies are required to show that an infant formula with a protein content of less than 2 g/100 kcal is suitable for the particular nutritional use of infants.

Against this background the primary objective of this study is to show non inferiority of an infant formula with a protein content of 1.89 g/100 kcal compared to a standard infant formula with a protein content of 2.2 g/100 kcal referring to growth parameters of healthy term infants receiving this formulas fully during the first 4 months of life after establishment of full formula feeding no later than postnatal day 28.

The secondary objective of the study is to show that parameters of protein status (serum albumine, blood urea nitrogen, serum creatinine, plasma amino acid-profile) in blood of infants receiving the modified infant formula (protein: 1.89 g/100 kcal) instead of the standard formula (protein: 2.2 g/100 kcal) are closer to that of fully breastfed infants. Furthermore fatty acid-profile will be assessed and compared between the groups.

4 Study Design

4.1 Type of study

The proposed study is a double-blind, controlled, monocenter, prospective, randomised, parallel design, intervention study. It will be conducted at the Ginekološko Akušerska Klinika “Narodni Front” Hospital for Gynaecology and Obstetrics in Belgrade (Serbia) under supervision of Prof. B. Koletzko (Chief Investigator), Dr. von Hauner Children’s Hospital / LMU.

4.2 Groups and subjects

For the proposed study three different groups have been defined:

**Group**	**Feeding (fully)**	**Formula**	**n**
Reference group	Breastfeeding	-	100
Control group	Formula-feeding	Conventional HiPP formula<br>protein content: 2.2 g/100 kcal	100
Study group	Formula-feeding	Modified Study formula<br>Protein content: 1.89 g/100 kcal	100

Based on power calculation 70 subjects have to complete the study in accordance with the protocol in each group. Since a drop out rate of 30% is estimated, 100 subjects / group needs to be recruited.

Assignment to the groups will be done at parents' / caregivers' option; depending on whether they want to fully breastfeed or fully formula-feed their infant until the age of 120 days.

If mothers decide to breastfeed their babies fully during the first 120 days of life, they can be included in the reference group. Children of mothers, who can not breastfeed their healthy newborn babies for reasons not related to this study, or who decided – in spite of all benefits of breast milk – to start fully formula-feeding within the first 28 days of life, will be randomised double blinded into one of the two formula groups.

4.3 Duration of subject participation

After a run in phase from Baseline Visit (day 4-5 of life) until the age of 28 days for the formulae groups (phase of change to the study formula), subject will be fully formula-fed until the age of 120 days. Infants of the reference breastfeeding group will be fully breast fed from birth until the age of 120 days. At 120 ±3 days of age the subject participation is terminated.

5 Study population

5.1 Description

Healthy, term newborns will be enrolled into the study. At the beginning of the study (Baseline Visit and recruitment) infants must not be older than 5 days. Randomisation can take place until the age of 14 days (parents wish) or later (before day 28) if the investigator is sure that the subject is able to shift to full formula feeding latest until day 28 of life. The study will end with the final Visit (Visit 4) at the age of 120 ± 3 days. Proportions of male and female subjects must be equal in both formula groups (stratification for gender).

5.2 Subject inclusion criteria

Subjects will be eligible for inclusion in the study if they meet all the following inclusion criteria:

- Healthy term newborn
- gestational age between 37 and 41 weeks
- birth weight between 3rd and 97th percentile of EURO GROWTH weight-for-age standards:
  - Girls: ≤ 2.640 and ≥ 4.203 g
  - Boys: ≤ 2.662 and ≥ 4.364 g
- age of enrolment: 4th or 5th day of life
- parental informed consent is obtained
- Serbian nationality (with belonging to sub-group specified)

5.3 Subject exclusion criteria

In general, infants with suspicion or prove of a systemic illness that may alter or influence metabolism must not be included in the study.
Subjects, who fulfil one or more of the following criteria are not eligible for study participation:

- Malformations, congenital heart defect, congenital vascular disease, severe diseases of gastrointestinal tract, kidney, liver, central nervous system and / or metabolic disease
- Intensive care after birth
- gestational age ≤ 37 and ≥ completed 41 weeks (Birth before 37 weeks + 0 days or after 41 weeks + 6 days of gestation)
- birth weight lower than 2.640 g (girls) / 2.662 g (boys) and higher than 4.203 g (girls) / 4.364 g (boys)
- age > 5 days of life at beginning of the study
- participation in any other clinical study intervention
- twins, multiple birth
- neonatal infection: suspected or confirmed sepsis, antimicrobial chemotherapy (or: other generalized infectious disease)
- medication and parenteral nutrition: other than supplementation of Vitamin K and D and vaccination (short post-natal treatment with intravenous saline or glucose solutions does not demand exclusion)
- metabolic disorders (inborn error of metabolism in infant or family member, gestational diabetes in mother)
- birth-related complications (e.g. asphyxia, meconium aspiration, others)

- o Severe disturbances of neonatal adaption: hypo- (<2.8 mmol/l = <50 mg/dl) / hyperglycemia (> 6.4 mmol/l), hypotonia (systolic <55 mm Hg), respiratory distress, hyperbilirubinemia (>300 µmol/l total bilirubin at any time)

The check of inclusion and exclusion criteria will be done at Baseline Visit and has to be documented in the Case Report Form.

5.4 Definition of Compliance

The investigator who informs the parents/caregivers about study details before the parents/ caregivers give their informed consent needs to explain that it is important to feed the infants in accordance with the study protocol. Infants in the breastfeeding reference group must be fully breast fed from day 0 until day 120 of age. Subjects of one of the study formula groups must be fully formula fed latest from day 28 until day 120.
In case infants are not fully breast or formula fed it is of great importance to document such deviations from the protocol in the CRF. The definition of what is fully breast fed and fully formula fed is given below (5.4.1). This definition should not be communicated to the parents/ caregivers but is for evaluation whether the subject was in accordance with the protocol or a non-compliance with protocol is given.

5.4.1 Definition of fully formula-feeding and fully breastfeeding

Fully formula-feeding is defined as:

Subjects must receive the respective study formula only, but…

- beneath study formulae breastfeeding once a day is acceptable
- Feeding of any other formula is not allowed
- Minor amounts of liquids: e.g. <50 ml unsweetened tea or water / day are allowed
- Minor amounts of complementary feeding: ≤ 3 spoons / week (e.g. fruit, vegetables, cereals, baby rice, lactose etc.) – are allowed

Fully breast-feeding is defined as:

Subjects in the reference group must receive breast milk only, but…

- Beneath breast milk formula feeding once a day is acceptable
- Minor amounts of liquids - e.g. <50 ml unsweetened tea or water / day - are allowed
- Minor amounts of complementary feeding - ≤ 3 spoons / week (e.g. fruit, vegetables, cereals, baby rice, lactose etc.) – are allowed

5.5 Definition of Non- Compliance

5.5.1 Severe non-Compliance (ITT Population)

Full analysis (Intention-to-Treat = ITT Principal) comprises all subjects who were enrolled including those with minor and severe non-compliance to protocol, subjects with missing values (but who participated at least in Visit 1) and protocol violations. Solely subjects turned out to be a “screening failure” (does not fit to an important inclusion or exclusion criteria) will not be considered for full analysis.

- Severe non-compliance with the study protocol or instructions
  - feeding does not comply with the above given definition of “fully” (e.g. implementation of weaning food before end of 4th month, great amounts of sweetened tea)
  - important missing values (infant was not weighed at Visit 1 or Visit 4), massive incomplete observation (no information about formula intake was received)
  - treatment with unauthorized medication
  - severe protocol violations (e.g. randomisation not according to randomisation list, informed consent was not received/signed, ignorance of documentation of data in the CRF, conduct of the study against specification of the study protocol).

All protocol violations/deviations need to be documented in the protocol violation form (Appendix 15.22). Deviations to the protocol are any difference that occurs in the study conduct whether it is in the criteria, the procedure, or devised approved protocol. A deviation does not affect the participant’s safety, rights, welfare, the integrity of the study, or its resultant data. Violations to the protocol are deviations that increase the risk, decrease the benefit and/or affect the subject’s rights, safety, welfare and/or the integrity of the resultant data. The Investigator needs to inform the Monitor and the Sponsor about protocol violations. In case of severe protocol violation the investigator also needs to inform the Ethical Committee by providing a report.

5.5.2 Minor non-compliance (per protocol population)

The Per-Protocol Population (PPP) is defined as the set of subjects without severe non-compliance to the protocol. Minor non-compliance e.g. visit outside the given window, or missing values of marginal importance (stool colour not reported once) are acceptable and do not lead to exclusion from the PPP analysis.

- Minor non-compliance with / Minor Protocol violations (deviations) of the study protocol or instructions
  - subject visit conducted outside of the visit window (±3 days)
  - anthropometric measures were not done in duplicate on some occasion (one or two visits)
  - missing values of minor importance (e.g. stool colour reported not on every diary etc.)

5.5.3 Subject drop out

If one or some of the following aspects will occur, subject will be defined as a drop out of the study:

- Lost-to-follow up: Relocation of family
- Withdrawal: Parents / caregivers wish
- Paediatricians recommendations to stop feeding the formula, due to a serious adverse event (incomplete treatment)
- Participation in another clinical trial

Detailed explanation of early drop out should be recorded in the CRF (Withdrawal sheet) with appropriate communication to the monitor.

Please note: if a subject is willing to join the study, but does not fulfil all inclusion criteria or an exclusion criterion applies before randomisation, this is not a drop out. These subjects will be recorded as non-eligible subjects.

6 Treatment of subjects

6.1 Product description

6.1.1 Composition

The following infant formulae will be used in the study:

1. Intervention Formula: Modified HiPP infant formula with a reduced protein content of 1.89 g/100 kcal and LCPs (arachidonic acid and docosahexaenoic acid, each 12.3 mg/100 kcal = 0.23%).
2. Control Formula: Standard HiPP infant formula with a protein content of 2.2 g/100 kcal, without LCPs. Commercial product.

Both infant formulae are in accordance with relevant EU-directives: for Intervention Formula the Directive 2006/141/EC on Infant Formulae and Follow-on Formulae is relevant. The Control Formula is in accordance with Directive 1999/21/EC. Both formulae are in accordance with Codex Alimentarius standard for Infant Formula.

The infant formulae consist of following ingredients:

Ingredients - Modified study formula

Skimmed milk, lactose, partially demineralised sweet whey powder, vegetable oils, LCP-mixture (egg oil, vegetable oils, fish oil), whey protein isolate, calcium chloride, potassium chloride, emulsifier soy lecithin, sodium citrate, L-phenylalanine, vitamin C, manganese carbonate, potassium salts of orthophosphate acids, taurine, L-thryptophan, inositol, iron sulphate, stabilizer L(+)-lactic acid, vitamin E, niacin, zinc oxide, pantothenic acid, copper-lysine complex, vitamin A, vitamin B_1, vitamin B_6, vitamin B_2, potassium iodate, manganese sulphate, folic acid, vitamin K, sodium selenate, biotin, vitamin D, vitamin B_{12}.

Ingredients - Standard Study formula (Control formula)

Partially demineralised sweet whey, skimmed milk, vegetable oils, partially demineralised sweet whey powder, lactose, calcium chloride, vitamin C, calcium carbonat, potassium chloride, iron lactate, zinc sulphate, niacin, pantothenic acid, vitamin E, copper sulphate, vitamin A, vitamin B_1, vitamin B_6, potassium iodate, folic acid, vitamin B_2, vitamin K, biotin, vitamin D.

For detailed description of the composition per 100 g powder, per 100 ml ready to drink product and 100 kcal: see Appendix 15.1 and 15.2.

The casein/whey ratio (40% casein, 60% whey) as well as the casein source (skimmed milk) is identically in both formulae.

The vegetable fat mix in the standard formula is composed of: palm oil, rapeseed oil, sun flower oil. This leads to the following fatty acid composition: 36.2% saturated fatty acids, 40.7% monounsaturated fatty acids and 23% polyunsaturated fatty acids (of which are: 90,2% linoleic acid, 9.8% linolenic acid).

The vegetable fat mix in the intervention formula is composed of: palmolein, rapeseed oil, palm kernel oil, sun flower oil. In addition to the vegetable oils, the intervention formula contains a LCP- mixture with the following composition: egg oil, vegetable mix as described above, fish oil. The proportion of fatty acids in the intervention formula is as follows: 35.4% saturated fatty acids, 43.7% monounsaturated fatty acids and 20.9% polyunsaturated fatty acids (of which are: 86,2% linoleic acid, 10.3% linolenic acid, DHA and ARA each 1.1%).

The used infant formulae differ according to following aspects (theoretical values derived from calculation based on the recipe):

	**Intervention Formula (modified)**	**Control Formula (standard)**
**Energy** (kcal/100 g)	518	513
**Protein** (g/100 kcal)	1.89	2.2
**Manganese** (µg/100 kcal)	10	--
**Selenium** (µg/100 kcal)	2.3	--
**Inositol,Taurine**	added	--
**LCPs**	enriched with arachidonic acid and docosahexaenoic acid from fish- and egg-oil each 0,23% - 12.3 mg/100 kcal	--

6.1.2 Form and dosage

Both infant formulae are available as powder.

The preparation will be carried out as described on the package with the separately provided spoon (Appendix 15.3). 8 of these spoons, put together in a closed envelope labelled with the respective Study Formula Random number, will be provided to the parents together with the formula at Baseline Visit and Visit 2. Separately provided spoons are light blue in contrast to the original dark blue spoons. To make sure that the parents/caregiver used the right spoons usage of light blue spoons will be asked for in the diaries. In addition, the parents/caregivers will be reminded to use these spoons at every Visit and Reminder Call by the investigator.

6.1.3 Quality control and shelf life

Quality control is performed by HiPP at the stage of raw materials, during production and on product release. The shelf life of the product is 15 months (modified study formula) and 18 month (control formula), respectively. A best before date is printed on each package.

6.1.4 Packaging and labelling

Study formulae are packed in white 600g-paper-boxes. Each box contains two bags (a 300 g) and a measure scope. Four of these paper-boxes are put together in a covering box (4 x 600g-boxes).

Declaration on the 600g-paper boxes is done in English and Serbian (Appendix 15.3). The study formulae are named as "HiPP PRE Infant Milk formula / from birth onwards" / "HiPP PRE Mlecna formula za mladu odojcad / Od rodenja nadalje".

6.1.5 Blinding technique

All study formula will be blinded by the sponsor according to study design. The products will be packed in identical white boxes and labelled with the same product name. The different standard solution is not written on the package - to achieve an equal dosage table, different spoons will be provided for each subject.

The identity of the specific product will be blind to subjects, support staff and investigators.

Formula boxes and spoon packages are marked with specified Study Formula Random Number (chapter 9.6).

6.2 Treatment administration

6.2.1 Amount, dose, frequency

Parents will receive first formula boxes at Baseline Visit if the infant is randomised at this visit. If not the parents will receive formula boxes after randomisation. Change from another formula or from breastfeeding to fully study formula-feeding must be carried out until beginning of Day 28 (run-in phase). Per definition Day 28 is the latest possible day to fully feed the study formula for the first time. Fully formula feeding must start at that day and then continue until Visit 4 (120 ± 3 days of life).

In case the healthy infant is not breastfed, control formula as well as modified study formula is suitable to be fed from birth onwards.

Study formulae must be given fully as sole nutrition (ad libitum). A feeding guide on the packages will give parents / caregivers advice regarding the average drinking amounts and bottle feeds per day (Appendix 15.3). As this is a guide only a baby may need more or less

than the volumes stated. In this case consultation of the study paediatrician is recommended. The exact amounts consumed will be written down in the diary.

6.2.2 Route of administration

Study formulae will be given in baby bottles (glass or plastic) prepared according to the procedure described on the label (Appendix 15.3).

6.2.3 Subject compliance

The parents / caregivers are asked to bring back all empty formula boxes always to the next visit. For the last Visit (Visit 4) they are asked to bring back the empty and the unused formula boxes. After study termination the formula not used will be destroyed in Belgrade. Date of destruction must be documented in the study formula inventory form.

6.3 Concomitant diet and treatment

6.3.1 Concomitant diet

As defined in chapter 5.4.1 subjects must be fully breast-fed throughout the whole study period (from day 0 to day 120 ± 3 of life) or fully formula-fed latest before day 28 until completion of the study (120 ± 3 days of life). Partial breastfeeding or feeding with another formula is only acceptable in the formulae groups within the run in period (from birth until Day 27).

Parents are asked, according to national Serbian recommendations and in accordance with ESPGHAN recommendations (ESPGHAN Committee on nutrition: Agostino et al. Complementary Feeding: A Commentary by ESPGHAN Committee on Nutrition; JPGN, 46:99-110, 2008), not to start complementary feeding before the age of 17 weeks (Visit 4).

Minor amounts of unsweetened tea or water are acceptable. Regular feeding of sweetened tea, complementary food, other infant formulae or addition of any kind of supplement to the formula (e.g. lactose, baby rice, cereal) is a major non-compliance with the protocol (see Chapter 5.4 and 5.5).

In any case concomitant diet must be reported in the diaries, the reminder calls and the CRF as “kind of diet”, “frequency” and “amount”.

6.3.2 Concomitant treatments / medications

All concomitant medications / treatments as well as the underlying diseases must be reported in the CRF. Vitamin K, Vitamin D and treatment of reaction on vaccination (suppositories) are permitted. Treatment of mild forms of infections, e.g. mild infections of the respiratory tract treated with inhalation of NaCl-Solution, is permitted.

Antibiotic treatment of the child is an unauthorized treatment with regard to study conduct as well as treatment e.g. with corticosteroid-containing medications. In addition, treatment of

severe infections (e.g. pneumonia, severe diarrhoea with vomiting, requiring hospitalisation > 3 days) is an unauthorized treatment with regard to study conduct.
In any case medication during study conduct must be reported in the Case Report Forms.

6.4 Product handling

6.4.1 Distribution and Storage

All study formula will be supplied by HiPP without charge to the study site and will be stored at *alca trgovina d.o.o.m,* Belgrade. The study formula will be supplied to study site according to need. Supply of study formula must be documented (amount, dated and signed) in the study formula inventory form (Appendix 15.4). Study staff in the hospital will hand out the formula to the parents / caregivers that gave informed consent to participate in the study according to the Study Formula Random Number. Mistakes in the distribution of the formula have to be excluded and accountability must be reported in the Study Formula Accountability Form that is part of the CRF (Appendix 15.12).
During the whole study each subject will receive a total of 24 x 600gr-boxes (= 6 covering boxes) with his or her own specified Study Formula Random Number. At Baseline Visit and Visit 2 they will receive 2 boxes and a separate random number labelled envelope with light blue spoons, and on Visit 1 and Visit 3 they will receive one covering box. In case parents / caregivers ask for more formula, study team has to follow this request and document it in the Study Formula Accountability Form.
If a subject withdraws, no more study formula is dispensed to this subject.

The powder has to be stored at a dry, not too warm place (room temperature) and has to be closed carefully. It has to be used within three weeks after opening the bag.

6.4.2 Product accountability and reconciliation

All study formula received by the investigator must be inventoried and accounted for throughout the study period on the corresponding Study Formula Inventory Forms (Appendix 15.4) by the study team in Belgrade. The dispensed study formula to the subjects must be inventoried and accounted for throughout the study period on the corresponding Study Formula Accountability Form that is part of the CRF (Appendix 15.12).
These two forms will be monitored.

The study formula must not be given to any person except the study personnel and subjects participating in this study.

Unused formula must not be traded. Infant formula which has not been used by the parents / caregivers has to be given back to the investigators. At the end of the study, all used and unused study formula must be accounted for on the Study Formula Accountability Forms as well. Unused products left in the hospital can be returned via the HiPP representative in Serbia for destruction or the hospital can arrange for its destruction at the end of the study. Destruction must be documented in the study formula inventory from.

7 Definition of measures

7.1 Primary Outcome

The primary outcome of the study is the average daily weight gain in grams (g/d) between Visit 1 (30 ± 2 days of life) and Visit 4 (120 ± 3 days of life).

The weight in grams will be assessed at each Visit by responsible study person and average daily weight gain in g/d during the study period will be calculated.

7.2 Secondary Outcomes

7.2.1 Anthropometric data

In the context of all paediatricians´ examinations anthropometric data such as length (cm) and head circumference (cm) will be recorded.

7.2.2 Protein status measurements

At Baseline Visit and the final Visit 4 blood will be drawn to assess protein status and metabolism with following parameters:

- serum albumin
- serum creatinine
- blood urea nitrogen
- plasma amino acid profile (Appendix 15.15)

7.2.3 Fatty acid profile in plasma

At Baseline Visit and the final Visit 4 blood will be drawn and the profile of defined fatty acids will be analyzed (Appendix 15.16).

7.3 Other measures

7.3.1 Physical Examination

Status of the child will be examined in a clinical examination at every Visit. It will be determined if findings are normal or remarkable. The following will be covered:

- General appearance
- Skin
- Fontanel
- Ear/Nose/Throat
- Lymphoid nodules
- Heart
- Pulmo

  - Abdomen
  - Extremities
  - CNS
  - Others
  - Medications necessary

7.3.2 Formula intake

In the context of the Visits, the reminder calls and the diaries filled out at home, formula intake will be reported by assessing:

- Visits/Reminder calls: formula meals per day, breastfeeding meals per day, meals of others
- Diaries:
  - number of bottles per day
  - intake of formula in ml per meal (documentation of prepared formula in ml and leftover in the bottle in ml)
  - acceptance of formula (good / reluctant / bad)

In the breastfeeding reference group similar dairies will have to be filled in asking also for feeding history (number of breast meals per day).

7.3.3 Tolerance

In the context of the diaries filled out at home formula tolerance will be reported by assessing:

- Stool characteristics
  - stool frequency: >5/day (very often), >3/day (often), 1-2/day (regularly), <1/day (not often), less than every third day (seldom)
  - stool colour: yellow, yellow-green-brown (mustard), green, brown, grey, other
  - stool consistency: hard lumps, sausage with cracks, soft sausage, mushy (like porridge), watery

- Digestion history
  - Regurgitation / vomiting
    - Regurgitation is observed when formula which has been previously consumed is brought back up through the oesophagus into the mouth. It has to be accounted by the parent / caregiver into the diary
    - Vomiting is defined as the expulsion of the refluxed gastric content into the mouth (choke and gush). It has to be accounted by the parent / caregiver into the diary

- Colic
  - Colic is defined as an abdominal pain, manifested by severe crying, drawing the lags up to the abdomen and distended and/or tense abdominal wall.
  - Frequency during the past day is reported as: not at all, <3 hours/day, >3 day hours/day
- Flatulence
  - Flatulence is defined as excessive bowel air and related discomfort.
  - Frequency during the past day is reported as: not at all, < 3 hours, 3-6 hours, 6-12 hours, >12 hours / day

In the breastfeeding reference group similar dairies will have to be filled in asking also for stool characteristics and digestion history.

7.3.4 Compliance to study protocol

Compliance to the study protocol will be checked during each clinical study Visit and during the telephone interview. It must be made sure, that:

- No other Formula but the study formulae is given to the formula group infants
- **Full breastfeeding** or **full formula-feeding** (Definition see Chapter 5.4.1), respectively, is maintained
- The above mentioned aspect will be clarified by asking for breast milk meals per day, formula meals per day and any concomitant meals in the CRF
- Absence of any unauthorized concomitant diet (excessive additional liquid intake, complementary food)

7.3.5 Health observations

The paediatrician is requested to report health associated observations during the last 4 weeks (time period between the visits, reported by parents/caregivers) at each clinical study Visit. Health observations and -if used- medication must be documented in the CRF. Health observations are for example:

- nappy rash
- skin problems
- slimy stools
- infections

7.3.6 Formula associated observations

Formula associated observations will be recorded during each clinical study Visit and during the telephone interview:

- Formula acceptance of the subject should be reported as good / reluctant / bad.

If “reluctant” or “bad” is marked with a cross, the investigator should ask for the reason why and record the reason in the respective space in the CRF/Telephone interview protocol.

- Formula handling should be reported as good / moderate / bad

If “moderate” or “bad” is marked with a cross, the investigator should ask for the reason why and record the reason in the respective space in the CRF/Telephone interview protocol.

8 Conduct of the study

8.1 Study plan, flow chart

See Flow Chart (Appendix 15.4) and Time Event Schedule in the appendix (Appendix 15.6).

8.2 Time schedule

The expected duration of the study will be approximately 26 month. Four months will be needed for preparation of the documents, study organisation and study initiation at study site. At the beginning of June 2009 the first subject will be enrolled in the study. Recruitment of the subjects is estimated to take 12 months (1 year). The latest subject will finish the study 16 month after start of recruitment. After termination of the study, another 6 month will be needed for laboratory analyses, evaluation of the findings and writing the final study report.

Time schedule of duration

month	1	2	3	4	5 - 20	21	22	23	24	25	26
activity	Document preparation										
		Study organisation and initiation at Site									
					Recruitment of subjects + intervention						
						Labor. analyses + Evaluation					
									Final report / Publication		

8.3 Study initiation Visit

Prior to study start, the entire study team will be educated by the Monitor regarding the study design and study requirements during a study initiation Visit. The Principal Investigator is responsible to train new study staff members according the study protocol. All people that are delegated by the Principal Investigator to conduct specific duties during the BeMIM study must sign the Signature Sheet. The name of the delegated person as well as the conducted duties must be written in this sheet.

8.4 Parent information, Subject recruitment and Randomisation

Parents / caregivers will receive information about the study before birth or within the first few days after birth (from birth to the 5th day of life) at the study site from a member of the study team. In case of interest, parents / caregivers will get contact information and are asked to get in touch with the study team. In compliance with the CONSORT statement the number of subjects assessed for eligibility will be reported (Subject Screening list).

Shortly before Baseline Visit parents / caregivers will receive Parent Information and an Informed Consent form from the study team (Appendix 15.7; Appendix 15.8).

The informing conversation should be made by educated study staff, the paediatric physician primarily involved in conducting the study or by a colleague paediatric or obstetric physician that is familiar with the study design and is able to answer parents / caregivers questions in detail. During the parent information conversation the investigator should clarify whether the infant will fit to the inclusion and exclusion criteria (as described in the “parent information”) to prevent unnecessary non-eligible subjects signing the informed consent. The Principal Investigator must clarify if parents / caregiver are able to understand and speak serbian language fluently to make sure they will understand all aspects relevant for them.

Subjects are recruited, if their parents / caregivers gave written informed consent and if they had been successfully screened for inclusion and exclusion criteria (chapter 5). The phase of recruitment covers the period of day 4 or 5 of life. Subjects recruited for the study will be assigned to a four-digit Subject ID Number starting with a zero (e.g. 0001 – 0300). Assignment to the Subject ID Number is documented on the “Subject Screening List” (Appendix 15.9). The Subjects ID number needs to be transferred to the “Subject Identification List” (Appendix 15.10) as well as the “Subject Randomisation List” (Appendix 15.11). Contact data will be documented on the Subject Identification List. The latter will remain at the study site and may not be forwarded to the sponsor (data protection).

The Subject ID Number will accompany parents / caregivers and subject throughout the study duration. All blood samples as well as all diaries and Visit documentation sheets will be coded with this Subject ID Number.

Subject finally assigned for the formula groups will receive a Formula Random Number as well (chapter 9.6). This number will not replace the Subject ID Number. The Formula Random numbers are 3 digit numbers which will be assigned in ascending order, for girls starting with 101 continuing to 199 and going on with 301. For boys starting with 201 to 299 and proceeding with 401. All subjects finally enrolled (including breastfed infants without Formula Random number) will be documented without contact details in the “Subject Randomisation List”.

Randomisation will be done at best at the Baseline Visit, but can be done until day 14 if parents/caregivers decide -due to any reason not related to this study- to skip breastfeeding during this time. All mothers will be provided with the information that if they against all recommendations decide to stop breastfeeding their infant can be randomised into one of the study formula groups until day 14 of life. Later on they might also be randomised based upon decision of the Principal investigator when the Principal investigator makes sure that mother will be able to fulfil a complete change to study formula not later than day 28.

The Principal Investigator and his study team will do all efforts to enrol a total of 300 subjects within one year – but within 1 ½ year the latest.

8.5 Visit

8.5.1 Baseline Visit (4-5 days of life)

The Subjects ID Number and date of Visit must be reported on the CRF (Baseline Visit).

8.5.1.1 Inclusion and exclusion criteria

After written consent was given from the parents, inclusion and exclusion criteria (see 5.2 and 5.3) will be assessed and documented in the CRF (Appendix 15.12).

8.5.1.2 Core data

Following core data parameters will be assessed at Baseline:

Core Data

- Infant Core data
  - Sex
  - Date of birth
  - Gestation in completed weeks
  - Mode of delivery
  - Birth weight
  - Body length at birth
  - Head circumference at birth
  - APGAR score
  - Care of child (mother, daily nursery,…)

- Core data parents

  Mother (only)
  - Date of birth
  - First child
  - Smoking
  - Maternal education (Basic – additional – tertiary)

  Mother and father
  - Weight before pregnancy
  - Weight at the end of pregnancy (before birth; mother only)
  - Weight and height at Recruitment
  - Diagnosed Allergies
  - Ethnicity (Country of birth, cultural heritage)
  - Socioeconomic status (measured by occupation)

Feeding history from birth until recruitment

Following aspects concerning the feeding from birth until recruitment must be documented:

- Type of feeding at recruitment: formula-feeding, breastfeeding
- Duration of exclusively breastfeeding
- Duration of partial breastfeeding
- Duration of exclusively formula-feeding
- Type of formula used so far (brandname)

8.5.1.3 Anthropometric data

Anthropometric measurement must be performed within the paediatrician's examination as described below (SOP for anthropometric measurements). Always the same paediatrician or member of the study team has to perform this measurement. To assure that variances through different methods of determination are minimized, study team members in the clinic will be trained together at the beginning of the study.

- o Body weight (g): measurement will be done with a calibrated scale (seca 336) with a precision of 10 g. The infant, without diaper, is placed on the scales so that the weight is equally distributed on each side of the center of the pan. Weight is recorded, to the nearest 10 g, with the infant lying quietly, which may require patience. The measurement is repeated and recorded twice after excluding any clearly erroneous results.

- o Body length: distance crown - sole (cm): Two observers are required to measure recumbent length. The subject lies in a supine position upon seca 232 length. The crown of the head touches the stationary, vertical head-board, and the center line of the body coincides with the center line of the measuring table. The infant's head is held with the Frankfurt Plane aligned perpendicular to the plane of the measuring table. The shoulders and buttocks are flat against the tabletop, with the shoulders and hips aligned at right angles to the long axis of the body. The legs are extended at the hips and knees and lie flat against the tabletop, with the arms resting against the sides of the trunk. The measurer positioning the head stands behind the end of the table to ensure that the subject does not change position and to check the alignment of the body with the long axis of the table. The second measurer makes sure both head and feet touch the headboard while the legs are straightened without forcing them. He places one hand on the knees to ensure that the legs remain flat on the table. He or she applies firm pressure with the other hand to shift the movable board against the heels. The measurement is repeated and recorded twice after excluding any clearly erroneous value. The length is recorded to the nearest 0.1 cm

o Head circumference (cm): An infant is measured when recumbent or seated. For this measurement is used an Insertion tape. The measurer stands facing the left side of the infant and places the tape so that the zero end is on the lateral aspect of the head. This involves passing the tape around the head and then transferring the ends of the tape from one hand to the other so that the zero mark on the tape is inferior to the value to be recorded. The tape is positioned so that large amounts of cranial hair (braids) are excluded. Anteriorly, the tape is placed just superior to the eyebrows and posteriorly it is placed so that the maximum circumference is measured. The plane of the tape must be the same on both sides of the head. The tape is pulled tightly to compress hair and obtain a measure that "approximates" cranial circumference. The measurement is recorded to the nearest 0.1 cm. The measurement is repeated and recorded twice after excluding any clearly erroneous value.

8.5.1.4 Physical examination

At the Visits paediatricians will perform a physical examination. It will be determined if findings are normal or remarkable. The following will be covered:

- General appearance
- Skin
- Fontanel
- Ear/Nose/Throat
- Lymphoid nodules
- Heart
- Pulmo
- Abdomen
- Extremities
- CNS
- Others
- Medications necessary

The paediatrician will also ask for side effects or any adverse effects or unwanted incidences. If the child is sick, diagnosis will be recorded as well as a comment / remark of the paediatrician.

8.5.1.5 Blood withdrawal

In the context of blood sampling following aspects will be reported:

o Child fasted (last feeding at least 2 hours ago)

o Time of last feeding (hours)

- o Amount of last feeding: complete meal / minor amounts
- o Blood samples taken
- o Enough blood taken
- o Blood analyses results

Blood count analysis result must be filled in the CRF after completed analysis. Original reports must be attached after last page of the CRF.
Results from Munich (amino acid profiling, fatty acid profiling) will be reported in one table for all subjects. This list will be attached to the file where CRF are stored after completion of analyses.

8.5.1.6 Present feeding

The paediatrician will ask for present feeding as:

- o Fully breastfeeding
- o Partially breastfeeding
- o Fully formula-feeding

According to the answer given by the parents / caregivers the paediatrician has to decide how to go on with the Visit. In case of fully formula-feeding and partially breast-feeding (potentially skipping breastfeeding) the child will be randomised.

8.5.1.7 Randomisation

If the present feeding is fully formula-feeding or partially breast-feeding (potentially skipping breastfeeding), the Visit can go on with Randomisation (chapter 8.4, chapter 9.6). Infants will be assigned to a Formula Random Number.

8.5.1.8 Formula accountability

Parents / caregiver will receive instructions on how to prepare the formula, two covering boxes with formula with their specific Formula Random Number and an envelope also labelled with the assigned Formula Random Number containing light blue spoons, to be used for preparation of the formula.
Hand out of formula and spoons must be documented in the Formula Accountability Form (FAF). The parents / caregivers will be told to bring back empty formula boxes at every visit.

8.5.1.9 Reminder (Diary)

Parents of randomised infants and parents of infants who are fully breastfed will receive the Diary 1 with the corresponding Subject ID Number and are asked to bring it back to the next Visit. Instructions on how to fill it out correctly are given on the diary. Diaries for the breast

feeding group and the formula feeding group differ slightly. The investigator needs to be aware of the two different types of diaries (Appendix 15.13).

8.5.1.10 Reminder (Visit)

At the end of the Visit an appointment for the next Visit will be made and the Investigator has to sign the CRF.

8.5.2 Visit 1 (Day 30±2), Visit 2 (Day 60±3) and Visit 3 (Day 90±3)

Visit 1, Visit 2 and Visit 3 will each be conducted in an equal manner as set out below. The Subjects ID Number and date of Visit must be reported on the CRFs. Anthropometric data will be assessed and a physical examination will be performed as described in chapter 8.5.1.3 and 8.5.1.4. At each Visit the Investigator n must check whether a case of "Withdrawal" is apparent or not. Is that the case the Investigator needs to complete the "Withdrawal sheet" at the end of the CRF.

8.5.2.1 Health observations

In addition any health observation observed during the period between the visits will be thoroughly documented on the CRF and if necessary documented in the respective form (e.g. AE).

8.5.2.2 Compliance to protocol

The compliance of subjects to the protocol will be checked according to following questions:

- Only assigned formula was given?
- Fully breastfeeding
- Fully formula-feeding
    - At Visit 1 only: if YES – indicate the date when for the first time fully formula-fed
- Liquid intake
- Complementary feeding

8.5.2.3 Formula associated observations

The paediatrician will ask for formula acceptance and formula handling.

8.5.2.4 Adverse / Serious adverse events

Adverse events (AE) and serious adverse events (SAE) must be well documented. AE needs to be documented in the “Non-serious AE” form attached to the CRF. SAE documentation and report need to follow another procedure (separate SAE form). The definition and handling of AE and SAE is described in chapter 10 .

8.5.2.5 Formula accountability

Subject of the control and study group will receive new study formula with their specific Random Number (Visit 1 and Visit 3: one covering box; Visit 2: two covering boxes and a numbered envelope with spoons). Hand out of formula and spoons must be documented in the Formula Accountability Form (FAF).

8.5.2.6 Reminder (Dairy)

Subjects will receive the next diary. Subjects of the reference group will receive the Diary (type “breast feeding”). The last Diary will be discussed with the paediatrician and missing details will be discussed and if necessary and possible completed.

8.5.2.7 Reminder (Visit)

At the end of the Visit an appointment for the next Visit will be made and the Investigator has to sign the CRF

8.5.3 Visit 4 (Day 120 ± 3)

Visit 4 will be performed similar to Baseline Visit with assessment of anthropometric data, a physical examination and blood withdrawal, but without core data assessment.

Like on the other Visits compliance to protocol will be assessed and formula associated observations reported. Adverse and Serious adverse events have to be documented as set out in Chapter 8.5.2.4.

The paediatrician has to discuss the last Diary 4 with the parents / caregivers.

8.5.3.1 Study termination

All subjects, who had been enrolled in the study according to inclusion and exclusion criteria and who took part in the study according to study protocol, will terminate the study regularly with the last Visit at the age of 120 ± 3 days (Visit 4). The study will be terminated when the last subject completed the last visit (“last subject out”). Not later than 90 days after termination of the study the ethical committee (EC) must be informed about study termination by the

Principal investigator. Not later than 1 year after study termination the principal investigator will provide a final study report to the EC if desired by the EC.

The paediatrician has to document if:

- the subject took part in the study successfully in accordance with the study protocol
  - if NO: the PI must check if a protocol violation was given and needs to complete the protocol violation/deviation form
- all of the formula boxes (empty and unused ones) are given back

Information about complementary feeding will be handed out to the parents together with a selection of baby products.

8.6 Diaries

Three days before each Visit (except Baseline Visit) parents / caregivers are asked to fill out a diary at home. Diaries for the formulae groups and the reference breastfeeding group differ slightly (Appendix 15.13).
Parents / caregivers of the formulae groups are asked to write down bottle feeding, concomitant diet and tolerance as defined in chapter 7.3.1. Parents / caregivers of the breastfeeding group are asked to write down number of breast meals, concomitant diet and tolerance.
Diaries will be brought back to the paediatrician at the next study Visit. It will be checked for correct filling out and if necessary entries will be discussed.

8.7 Reminder call

Between the Visits, parents / caregivers will be called by the study team at Day 15 (± 3), Day 45 (± 3), Day 75 (± 3) and Day 105 (± 3).
Following measures will be assessed and documented on the "protocol of the reminder call" (Appendix 15.14):

- Compliance to protocol (Type of feeding, Formula shift completed – if YES: first day of completely bottle feeding (Call 1 only), liquid intake, complementary feeding)
- Formula associated observations (acceptance, handling)
- Reminder (next visit, filling out the diary, empty formula boxes etc.)

If a member of the reference breast feeding group is called, non applicable questions/answers will be marked with NA (=not applicable) by the investigator on the protocol of the reminder call.

8.8 Biological samples

8.8.1 Responsibilities, parameters and methods

Following parameters will be assessed at study site in Belgrade:

For health evaluation of each subject:

- Full blood count with differential
- C-reactive protein (marker of acute infections)
- Astrup (marker for respiratory status)
- Electrolytes Na, K, Cl (electrolyte metabolism)
- Glucose
- Total bilirubin (Baseline Visit only)

For protein status assessment and estimation of metabolic burden caused by protein intake:

- Serum albumine
- Serum creatinine
- Blood urea nitrogen

Blood count will be analyzed by routine laboratory methods in clinical laboratory with Olympus analyzer. The same Olympus analyzer will be used for protein status measurements according to instruction manuals provided by Olympus GmbH, which are filled in the Trial Master File and Investigator Site File.

The group of Prof. Dr. Koletzko will conduct metabolome analysis including:

- plasma amino acid profile (protein status) by Liquid Chromatography-Tandem Mass Spectrometry (description in Appendix 15.15)
- plasma phospholipid fatty acid profile (metabolic status) by Gas chromatographic analysis with an Agilent 5890 II gas chromatograph (description Appendix 15.16)

In total an amount of ~4.7 ml of whole blood needs to be drawn at Baseline Visit and Visit 4 each. As back-up for metabolome analysis last blood drops from the plain needle will be collected on a filter card. Analyses regarding nucleic acids (DNA, RNA) will <u>not</u> be conducted with the samples obtained in the context of the BeMIM study.

The Principal Investigator will receive all analyses results to include them at the end of the CRF.

8.8.2 Collection and preparation

Some of the material for sample collection will be provided by the group of Prof. Dr. Koletzko, Dr. von Hauner Children's Hospital, Munich. A list of this material is added below (Appendix 15.17).

8.8.2.1 Time of sample collection

As far as possible, fasted blood samples should be collected - that means at Baseline Visit soonest 2 hours after the last feeding, at Visit 4 soonest 3 hours after the last feeding. Time (hours ago) of last feeding as well as amount of the last meal (complete meal or minor amount) must be reported.

8.8.2.2 Procedure of blood sampling

Blood will be drawn off only in agreement with the parents / caregivers (part of IC). The paediatrician is responsible for this. Pricking two times is considered as being ethical – but more than two times is not allowed. Procedure of blood sampling does not differ between Baseline Visit and Visit 4.

Preparation: Mark material for each subject identically (label will be provided by the LMU team) so that identification can be done easily:

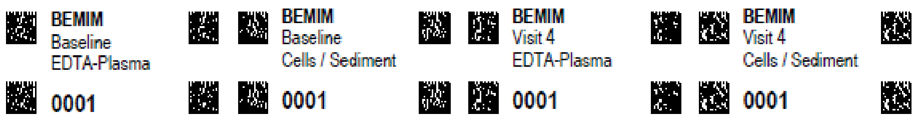

Volumes: A total of ~4.7 ml blood from a peripheral vein must be collected.

- *for analyses done in Belgrade:* a minimum of 2 ml blood must be collected in a standard tube(s)
- *for analysis done in Munich*: a minimum of 2.5 ml blood from a peripheral vein must be collected into an opened 2.7 ml S-Monovette (containing potassium-EDTA as clotting inhibitor) and on a filter card using a plain needle (see Appendix 15.18).

Procedure of blood sampling:

- unscrew the cap of the *S-Monovette*
- remove the syringe plunger of the *S-Monovette* by moving it backwards until it snaps in and break it by moving it to the side
- use a plain needle to let the infant's blood drop into the S-Monovette
- close the *S-Monovette* with the screw cap
- drop blood from the needle on the provided filter card to fill one circle completely for back-up of metabolome analyses.

Immediate further handling of blood samples:

For analysis done in Belgrade

- Transfer the vial for blood count analysis as soon as possible to clinical laboratory
- Protein status measurement:
  - transfer the vial as soon as possible to the laboratory for separation of cells and plasma by centrifugation. Store the vial at room temperature until centrifugation. Time under room temperature should not exceed 60 min.
  - Centrifuge for 10 min at room temperature (20°C) with 2000 x g.

For analysis done in Munich (see SOP in Appendix 15.18)

- Shake the *S-Monvette* – gently, but thoroughly – immediately after finishing blood collection.
- Transfer the filled *S-Monovette* as soon as possible to the laboratory for separation of cells and plasma by centrifugation. Time at room temperature before centrifugation must not exceed 60 min.
- Centrifuge for 10 min at room temperature (20°C) with 2000 x g.
- Transfer the plasma into a *Nunc 1 ml CyroTube* (see Appendix 15.17).

  The final volume of plasma samples from infants must be <u>1.0 ml</u> minimum!
- Label and store *Nunc 1 ml CryoTube* with plasma at -20°C.
- After removal of plasma and labeling, freeze the *S-Monovette* with the cells in an upright position at -20°C.
- After labeling, allow the *filter card* to dry at air for at least 4 hours. Then put it in the *plastic bag* and freeze at -20ºC.

8.8.3 Logistics

Plasma samples, frozen erythrocytes and filter cards for Munich will be collected and stored in a refrigerator at -20°C. The shipment to Munich will take place in regular intervals. Samples will be sent by express (DHL or Federal express) in isolated boxes on ~10 kg dry ice.

The study team in Belgrade is asked to inform members of Prof. Dr. Koletzko laboratory by telephone, Email or fax prior to shipment. Shipment should take place always on Mondays.

8.8.4 Reporting of results

Some free space is provided in the CRF of Baseline Visit and Visit 4 to fill in the results after complete blood analyses. Original reports must be attached after last page of the CRF. Results from Munich (amino acid profiling, fatty acid profiling) will be provided in a tabular form

comprising results of all infants. A copy of this comprehensive table will be attached in addition to the collected CRF's at the end of the study.

8.9 Breaking the code

The investigator will receive a set of sealed envelopes containing product identification.
The envelopes may be used in circumstances when the investigator identifies an urgent need to break the code. In such cases the rationale must be documented on the corresponding envelope, with immediate notification of the monitor.
The code break will also be reported in the CRF and events leading to the emergency breaking will be recorded in the serious adverse events report form. Code Break must also be documented in the protocol violation form (Appendix 15.22) as this is defined as a protocol violation.

8.10 Data management

8.10.1 Data on CRFs

All collected subject data will be recorded in compliance with GCP standard in Case Report Forms (CRF) (Appendix 15.12). Data of laboratory analysis done at site and at the von Hauner Children's Hospital, Munich will be entered as soon as results are obtained.
Site must complete the CRF in a timely manner and the investigator must promptly review and sign the completed CRFs for each subject. The Principal Investigator is responsible for quality, consistency and completeness of the CRF data.
The original CRFs will be collected by the Monitor and provided to the Sponsor. The Principal Investigator as well as the Monitor will receive a copy. All case report forms (CRFs) will be kept secret and stored for ten years by the Sponsor. Data protection will be guaranteed.

8.10.2 Computerized data entry

Primarily responsible for data entry is the team of the Chief Investigator (LMU). At site (Clinic in Belgrade) data will be entered by Dr. Stanimirovic from the CRFs into a computer database (Microsoft, Excel). Data entry will be verified by the study monitor (LMU). Evaluation of the data will be done by the Statistician at the von Hauner Children's Hospital, Munich using Stata® Data analysis and statistical software.

8.10.3 Organization of data sheets

Tab. 1 overviews how storage of all data sheets involved in this study will be handled. The team in Belgrade is responsible to organize distribution of most of the data sheets to the

sponsor (e.g. via the monitor) and the monitor. The sponsor and the monitor must store the data sheets in the corresponding files.

DOCUMENTS	Responsible	ORIGINAL		CARBON		COPY	
		Given to	File in	Given to	File in	Given to	File in
Informed consent	PI	PI	ISF	SUB			
CRF	PI	S	TMF	PI<br>M	ISF<br>-		
Diary	PI	PI	ISF			S<br>M	TMF<br>-
Reminder call	PI	PI	ISF	M	-	S	TMF
Subject screening list	PI	PI	ISF			S	TMF
Subject identification list	PI	PI	ISF				
Subject randomisation list	PI	PI	ISF			S<br>M	TMF<br>-
Serious adverse events	PI	PI	ISF			S<br>M	TMF<br>-
Study Formula Inventory Form	PI	PI	ISF			S	TMF
Monitoring reports	M	S	TMF			PI*	ISF
Report of laboratory analysis (LMU)	M	M	-			S	TMF
Report of laboratory analysis (Belgrade)	PI	PI	ISF			S<br>M	TMF<br>-
Final study report	M<br>PI	M<br>PI	-<br>-	S	-		

Tab. 1: Overview of data-sheet organization

Abbreviations
PI: Principal Investigator / Belgrade:
ISF: Investigator site file / Belgrade
SUB: Subject
M: Monitor / LMU:
S: Sponsor / Pfaffenhofen:
TMF: Trial master file / Pfaffenhofen

*: Principal investigator receives follow up letters during the study.

8.10.4 Unblinding procedure

After the last subject completed the study, all analyses are done and all data are entered from the CRF´s into the computer data base, data will be checked for completeness and plausibility. Last step before statistical analyses will be the unblinding of the Randomisation code. Information of the Randomisation Code will be provided by the Sponsor to the Chief Investigator and Principal Investigator Teams after the data base is locked.

9 Statistical methods and data analysis

9.1 Statistical background

The study is proposed to show the non-inferiority of an infant formula with reduced protein content (1.89 g/100 kcal) in comparison to a standard infant formula (2.2 g protein /100 kcal). Primarily weight gain in g/day will be assessed.

Statistics include detailed description of all recorded data. Regarding the comparison of the two formula groups general, adequate statistic designs will be applied. Statistic analysis will be performed with the program STATA 9.2.

9.2 Hypothesis to be tested

H_0 (to be rejected): The mean daily weight gain in the intervention group (formula with reduced protein content) is more than 3 grams per day lower than in the control group (standard formula).

$H_{alternative}$: The mean daily weight gain in the intervention group (formula with reduced protein content) is not more than 3 grams per day lower than in the control group (standard formula).

9.3 Primary outcome variable

The main outcome variable “weight gain” will be calculated by substracting the weight obtained at the 4 months study visit from the Visit 1 weight divided by the days passed between those visits.

9.4 Secondary outcome variables

Length gain: same calculation as for primary outcome

Gain in head circumference: same calculation as for primary outcome

9.5 Sample size calculation

Based on previous publications[14,15, 16, 23] and own data[6] we can expect the children to grow on average 30 grams per day with a standard deviation of 6 grams between birth and 4 months of life. Growth studies should be designed to have a power to detect a difference in weight gain equal to 0.5 standard deviations according to the SCF report[22]. Thus, one would need about 70 children in each formula group under the assumption of non-inferiority (one-sided test), a

significance level of 2.5% and a power of 85%. We expect a true loss-to follow-up of about 10-15%. Additionally, we expect that another 15% will switch formula due to preference changes which usually take place in the first months of life. These children will be only available for an intention-to-treat analysis. Thus, 100 children in each formula arm, as well as in the breastfed group will be included to have also sufficient power in a per-protocol analysis.

9.6 Randomisation

The Study Formula Random Numbers are computer generated to assure an equal and random distribution of study formula to the two different formulas helping to eliminate bias that could be introduced through a selective placement of study formula.
The study formula randomisation will be a permuted-block design to achieve equal numbers in each group. A block size of four will be used. The randomisation is based on sex-specific random list to achieve stratification for gender. The random lists will not be visible to the study personnel involved in recruitment, clinical appointments, sample collection, laboratory analyses and data evaluation.
Study Formula Random Numbers contain 3 digits and are sex-specific:

- Numbers for **girls** start with (uneven hundred number) 101 up to 199 and proceeding with 301
- Numbers for **boys** start with (even hundred number) 201 up to 299 and proceeding with 401

The investigator will receive a list with the ascending Random numbers for boys and girls that shall be followed carefully to assure data meet the assumptions necessary for statistical analysis (Appendix 15.23). If it is decided that the subject will be formula-fed, it will receive the next available study formula random number from the respective sex-specific random list. One by one consecutively Study Formula Random Number has to be given to the subjects. Used Study Formula Random Numbers should be scratched out on the Random number list.

The assignment of the Study Formula Random Number must be documented carefully in the Subject Randomisation List, the Subject Identification List as well as in the individual CRF.

Subjects assigned to the reference breastfeeding group will not receive a Study Formula Random Number; during the course of the study they will only receive a Subject ID Number. Subject ID Number is given to each enrolled subject after informed consent is received.

The Study Formula Random Number will **not** replace the Subject ID Number of the subject. All blood samples and Visit documentation sheets (CRFs) will still be coded by the Subject ID Number primarily. For subjects in one of the formula groups the Study Formula Random

Number will be recorded in addition to the Subject ID Number during all Visits following the randomisation plan ("RANDOM NUMBERB CHART" (Girls/Boys)). At the end of the study the kind of formula given to a subject can be identified with the Study Formula Random Number.

9.7 Management of subject withdrawal

Drop outs will not be replaced as a drop-out rate of 30% is already included in the power calculation. Subjects classified as Drop outs (definition see Chapter 5.5.3) must be documented in the "Withdrawal sheet" at the end of the CRF.

9.8 Data management

All clinical data will be recorded in CRFs to be incorporated later into an electronic database with double data entry. The results of the laboratory analyses will be transcribed from lab print outs by double entry into the same database.

Entered data will be checked on plausibility and internal consistency. For this purpose anthropometric measurements will also be expressed as z-scores relative to the growth standards of the World Health Organization for breastfed children[23]. Z-scores will be calculated using WHO programs (http://www.who.int/childgrowth/software/en/). Extreme weight, length and head circumferences as defined by large standard deviation scores will be discarded as implausible:

Weight-for-age z-score (zwei) zwei < -6 or zwei > 5,

Length-for-age z-score (zlen) zlen < -6 or zlen > 6,

Weight-for-length/height z-score (zwfl) zwfl < -5 or zwfl > 5,

BMI-for-age z-score (zbmi) zbmi < -5 or zbmi > 5,

Head circumference-for-age z-score (zhc) zhc < -5 or zhc > 5.

9.9 Statistical analysis

For all quantitative parameters determined a test for normal distribution will be performed according to Kolmogorov-Smirnov. If normal distribution can be assumed, mean and standard deviation will be given for these parameters. For non-normally distributed data median and interquartile range will be provided and data will be transformed appropriately prior to statistical analysis.

For the primary analysis breastfed children will not be included. They serve rather as a reference for the interpretation of the data collected in the formula groups.

The influence of the study group on the primary outcome will be tested using linear regression with baseline values as co-variables. Parameters, which might influence anthropometric development of the infant, e.g. gender, parental smoking and socio-economic status, will be tested for their effect on the outcome variable and, if appropriate, will be included as confounding factors.

Interim analyses are not foreseen as valuable results can not be expected from analysing the data from a limited number of subjects and an association of increased risk of occurrence of adverse events with any of the studied formulas is not conceivable.

Statistical analyses will be performed on the data collected from all infants enrolled into the study and randomised to one of the formulas (intention to treat analysis) and on the data collected from all subjects, who completed the study according to the protocol (per-protocol analysis). The per-protocol analysis is considered the primary analysis; the intention-to-treat analysis has confirmative character.

Full analysis (Intention-to-Treat = ITT Principal) comprises all subjects who were enrolled including those with minor and severe non-compliance to protocol, subjects with missing values (but who participated at least in Visit 1) and protocol violations. Solely subjects turned out to be a “screening failure” (does not fit to an important inclusion or exclusion criteria) will not be considered for full analysis.

The Per-Protocol Population (PPP) is defined as the set of subjects without severe non-compliance to the protocol. Minor non-compliance e.g. visit outside the given window, or missing values of marginal importance (stool colour not reported once) are acceptable and do not lead to exclusion from the PPP analysis.

9.10 Methods secondary analysis

For analysis of secondary outcomes the same methods will be used as for the primary hypothesis.

9.11 Complementary analysis

To confirm compliance with the study protocol energy intake and macronutrient intake as calculated from 3-day food protocols (“Diaries”) will be compared by t-test between both intervention groups.

Plasma urea, amino acid levels and fatty acid composition will be considered as the main blood parameters measured in children and will be compared between both intervention groups.

9.12 Responsibility of statistics

Statistical analyses will be supervised by Dr. Veit Grote, member of the team of the chief investigator Prof. Dr. Koletzko at the Dr. von Hauner Children’s Hospital, Munich.

10 Handling of Adverse events

10.1 Definition

10.1.1 Adverse event

The adverse event is defined as any untoward occurrence (including intercurrent diseases and accidents) in a patient or clinical investigation subject administered an investigational product and which does not necessarily have to have a causal relationship with the intervention.

Adverse events include occasions when the subjects contact the investigator or their private physicians and are examined or given medical direction. It may or may not lead to withdrawal of the subjects from the study. All adverse events occurring during the study will be reported and recorded whether or not they are considered to be non-serious or serious and related to the intervention.

10.1.2 Serious adverse event

A serious adverse event is defined as any adverse event that…

- requires hospitalisation or prolongation of existing hospitalisation
- is life-threatening
- results in permanent disability / incapacity
- results in death

10.2 Reporting and documentation

10.2.1 Reporting and documentation of adverse events (AE)

Documentation of Non-serious adverse events (AE) has to be done by the investigator on the corresponding page of the CRF during all visits and has to be classified as mild, moderate or severe (Appendix 15.12).

Documentation to include all of the following aspects:

1. History
    a) Diagnosis (if available; otherwise sign or symptom)
    b) Duration (start and end date of AE)
    c) Maximum intensity (mild / moderate / severe)
    d) Frequency
    e) Outcome
    f) Coherence to intervention formula

2. Action taken
   a) Change of intervention (no / reduction / stop)
   b) Therapy and treatment with drugs
   c) Withdrawal

Concerning "Outcome"

All AE must be followed until the events are resolved, the condition stabilises, the events are otherwise explained, or the subject is lost to follow-up. Indicate if the event was "recovered / resolved" or "recovered / resolved with sequelae". If the AE is ongoing at the time the subject completes the study or becomes lost to follow-up, the outcome must be recorded as "not recovered / not resolved" or "recovering / resolving". Confirm that these AE have been followed up for details of resolution.

Concerning "Frequency"

Select either "single episode" or "intermittent".

Intermittent should be used if the subject experiences the same AE on multiple occasions over a period of time. In these circumstances, the start date will be the start date of the first episode and the end date will be the date of resolution.

Concerning "Maximum intensity"

The maximum intensity of an adverse event is characterised as follows:

- *Mild*: short incidences which dose not cause special treatment and without influence on the clinical outcome of the subject
- *Moderate*: incidences which cause mild discomfort or problems and which could influence the clinical outcome of the subject a little. The discomfort can be treated easily.
- *Severe*: incidences which cause discomfort or problems and which have to be treated with drugs or which result in a therapy.

Concerning "Relationship to intervention"

A possible coherence between study formula and incidence has to be judged by the principal investigator. The following aspects have to be taken into consideration:

- Is there a temporal coherence between incidence and use of the study formula?
- Does a removal of study formula reduce the symptoms and do they get worse at re-introduction?
- Has the incidence been observed somewhere else, too?
- There is no coherence between incidence and other surrounding conditions, activities, or environmental factors?

Coherence between incidence and study formula is divided into:

- *no*: no coherence between incidence and study formula
- *weak*: less than 2 of the above mentioned criteria are fulfilled
- *possible*: at least 2 of the above mentioned criteria are fulfilled
- *likely*: at least 3 of the above mentioned criteria are fulfilled
- *definitely*: all 4 criteria are fulfilled

All AEs need to be followed-up. Those AEs that are not resolved until the last Visit (Visit 4) need to be documented in the AE follow up report. A copy of this report has to be sent to the monitor. A copy remains in the CRF.

10.2.2 Reporting and documentation of serious adverse events (SAE)

Documentation of a serious adverse event (SAE) requires that a separate Serious Adverse Event Form be completed in each case (Appendix 15.19). SAE must be reported immediately within 24 hours to the sponsor, the chief investigator and the monitor by phone and followed by faxing the filled in provided report that is part of the Serious Adverse Event Form. For the documentation it does not matter whether the incidence is associated with the study formula or not.

In addition to the above mentioned aspects for AE documentation, the documentation of a SAE includes:

a) Age of subject
b) Total intake of study formula on the day the SAE occurred first
c) Seriousness (specific reason for considering event as a SAE)
    - requires hospitalisation or prolongation of existing hospitalisation
    - is life-threatening
    - results in permanent disability / incapacity
    - results in death
d) Most likely cause of SAE
    - Disease under study
    - Others
e) Medical comments
f) Estimation of the Principal investigator whether there is an interrelation between the SAE and intervention, and if necessary: decision if the codes needs to be broken for this subject

All SAEs need to be followed-up, documented and follow-up needs to be reported to the sponsor, the chief investigator and the monitor.

11 Legal and ethical pre-requirements

11.1 Legal requirements

The study protocol will be accredited by the Ethical Committee in Belgrade / Serbia.

11.2 Ethical aspects

11.2.1 Protection of subject's confidentiality

Confidentiality of all study subjects will be maintained. Codes for subject's identification will be utilized. Documents that identify the subjects beyond Subject ID Number will not be submitted to the sponsor and must be maintained in strict confidence by the investigator, except to the extent necessary to allow monitoring by the study monitor or auditing by the regulatory authorities or an independent instance ordered by the sponsor.

11.3 Informed consent

The paediatrician will explain the purpose of the study in a way the parent / caregiver of potential eligible subjects are able to understand. A written information sheet in the native language of the parents / caregivers will be handed over (Appendix 15.7). All questions which will come up in the context of the study will be answered clearly and understandable. The parents of the subjects shall have sufficient opportunity to consider participation in the study. A parent of the subject cannot be led to believe that they are waiving their rights as a subject or the liability of the sponsor or investigator.

Before inclusion into the study a written informed consent has to be signed and dated by at least one parent or caregiver of the subject (Appendix 15.8) after information about the study. Single parents / caregivers need to sign without anybody else agreement. Acquisition of parent consent is the step by which subjects are enrolled into the study. Parents confirm that they have been informed properly and that the participation is voluntary. The Principal Investigator needs to point out that the parents / caregiver need to agree explicitly in taking of two blood samples. When signing the Informed consent parents / caregivers agree in taking of two blood samples (at the age of 4 or 5 days and at the age of 120 days) as mentioned explicitly on that form. Relevant information regarding the child which emerges due to the study participation will be presented to the parents / caregivers if they wish.

The informed consent has to be signed in two copies: one is kept in the subject's notes and one for the parents / caregivers.

11.4 Ethics committee approval

The study must not start before written consent is given by the Committee. If any changes or extensions are added after approval, the Ethic Committee has to be informed and approval must be given. If any severe adverse effects come up during the study period the Ethic Committee has to be informed.

11.5 Declaration of Helsinki

This study will be conducted according to the principles and rules laid down in the Declaration of Helsinki and its subsequent amendments (Appendix 15.20).

12 Quality assurance

12.1 GCP (Good clinical practice)

This study will be conducted according to the principles of ICH (International Conference on Harmonization) guideline for Good Clinical Practice* and the applicable regulatory or legal requirements. *(http://www.emea.europa.eu/pdfs/human/ich/013595en.pdf)

12.2 Internal Quality control

Internal quality control will be assured by Investigator meetings (including training on the protocol at site) and regular monitoring visits.

13 Agreements

13.1 Monitoring

The monitoring will be covered by the group of Prof. Dr. Koletzko, LMU Munich University Hospital (Martina Scheer). Monitoring needs to be conducted in accordance with the SOP for monitoring (Appendix 15.21) and in accordance with the recommendations "ICH – Note for Guidance on Good Clinical Practise" implemented in 1997 especially with Chapter 5.18 of the GCP Guidelines (ICH Topic E (6) R1 Guideline for good Clinical Practice, Step 5, Note for Guidance on Good Clinical Practice (CPMP/ICH/135/95).

During the study period a monitor will Visit the investigation group frequently and will check the Case Report Forms. The monitor will receive a study specific monitoring manual from the sponsor. The monitor starts visiting the Principal Investigator and its team before the study begins to exclude any insecurity regarding the study protocol or the procedure. In the following the monitor Visits the investigation team regularly. The monitor will check the ongoing of the study and the correct filling in of the Case Report Forms (CRFs). The CRFs will be signed by the Principal Investigator as well as by the monitor. After that they can be forwarded to the person who will be responsible for the data evaluation. At any time all data have to be available for the monitor as well as for the sponsor after request.

Recruitment progress will be checked a least every two weeks and progress needs to be reported to the sponsor.

After study initiation visit the monitor visits the site approximately every 5 weeks (~10 visits) in case recruiting proceeds as intended (recruiting terminated after 12 month).

Last Visit of the monitor will be as soon as the last child completed the study period. Every monitor Visit will be reported to the sponsor and a "Follow up" letter of identified deficiencies/mistakes, future strategies to avoid such deficiencies/mistakes and actions necessary to be taken by the site in a given time frame as mentioned in the monitoring report will be provided for the Principal investigator by the monitor. At the end of the trial the monitor will make a study closing visit to the site to ensure that all documentation is complete. Data protection issues have to be included into the monitoring.

13.2 Auditing

Auditors representing the sponsor my also similarly evaluate the study and its monitors. For these purposes, the Principal Investigator will make CRF and source documents available when requested.

In addition, the study may be evaluated by representatives of the national regulatory authorities, who will also be allowed access to study documents. The Principal Investigator must promptly notify the Sponsor of any audits they have scheduled with any regulatory authority.

13.3 Study report and publications

13.3.1 Study report

After termination (“last subject out”) of the study and as soon as the statistics are done, a study report will be written by the Chief Investigator (and team) in close collaboration with the Principal Investigator and after consultation of the Sponsor (HiPP GmbH). Not later than 3 month after study termination with completed analyses the study report needs to be finalised and provided to the sponsor.

13.3.2 Publications

Sponsor can use study results and reports before publication for internal purposes (including submission to the EFSA) and can cite published results without restrictions, respecting the publishers copyright. The Chief and Principal Investigator will do best efforts to publish the results in a scientific magazine.

13.4 Responsibilities

13.4.1 Overview

	Chief Investigator	Principal Investigator	Sponsor
**Study scheduling**			
Study protocol			x
Study documents			x
Subject Insurance			x
Ethical committee approval		x	
**Organisation**			
Supply of formula and logistics to Serbia			x
Logistic of formula in Serbia (store, use, dispose)		x	
Return / destruction of unused formula + reporting to Sponsor		x	
Monitoring	x		
Conduct of the study		x	
Provision of material and labels for biological samples	x	x	
Sample shipment to Munich		x	
AE and SAE reporting		x	
Data management	x	x	
Retention of records	x	x	x
Laboratory analysis	• Plasma amino acid profiling<br>• Plasma fatty acid profiling	Full Blood count with differential, CRP, Astrup, Electrolytes, Glucose, Serum albumin, Serum creatinine, Blood urea nitrogen	
Statistics	x		
Final study report	x	x	
Publication	x	x	

13.4.2 Responsibilities of the Principal Investigator

The Principal Investigator is in charge of the following procedures:

- Approval by the local Ethical Committee
- Recruitment of subjects in accordance with inclusion and exclusion criteria.
- Provision of informed consent signed by the parents / caregivers of the children
- Dispensation of study formula to the subjects
- Destruction and documentation of destruction of unused formula after study termination
- Compliance with the defined procedures in accordance with the "European Good Clinical Practice" (GCP)
- Treatment of all subjects in accordance with the protocol from start to study termination
- Complete and exact filling in of all Case Report Forms
- Organisation of sample shipment to Germany in coordination with the team at LMU Munich, Germany.
- Good cooperation with the monitor. The monitor has to be provided with original data, which are treated confidential.
- Data entry from CRF into computerized data base
- Announcement of all serious adverse events to the sponsor (HiPP GmbH) and the Chief Investigator (Prof. Dr. Koletzko).
- Retention of records: storage of all original data after completion of the study report for 15 years at the appointed location
- Confidential handling of all information coming up in the context of the study.
- Correct use of goods and money made available for the study.
- A deputy can be nominated by the Principal Investigator. Nevertheless the main responsibility stays at the Principal Investigator.
- Generation of final study report and publication in coordination with the Chief investigator.

13.4.3 Responsibilities of the Sponsor

- Supply of study formula in sufficient amounts free of charge
- Supply of incentives for all subjects that terminate the study in accordance with the protocol
- Supply of study protocol and all necessary documents
- Announcement of serious adverse events effects according to the national law (Serbia; Ethical committee)
- Insurance coverage in accordance with the existing law
- Payments as agreed on in the respective agreements
- Retention of records: storage of all original data after completion of the study report for 15 years at the appointed location

13.4.4 Responsibilities of the Chief Investigator (LMU) and team

- Study supervision
- Study management / support of study organisation at the site in Belgrade (GAK "Narodni Front" Hospital for Gynaecology and Obstetrics, Belgrade, Serbia)
- Monitoring
- Primarily responsible for data entry, data management and data evaluation (might be done in coordination with the team in Belgrade)
- Verification of sufficiency of documentation
- Statistics
- Laboratory analysis: plasma amino acid profiling, fatty acid profile
- Provision of a study report within 3 months after all analyses done in coordination with Principal Investigator
- Publication of results in a scientific magazine in coordination with Principal Investigator if suitability (right quality) of data is given.
- Retention of records: storage of all original data after completion of the study report for 15 years at the appointed location

13.4.5 Abnormal termination of the study

If the study has to be stopped, the Principal Investigator as well as its team will be informed in a written form. All documents related to the study will be sent back to the sponsor. Study formula will either be sent back to the sponsor or will be destroyed. In case of abnormal study termination the ethical committee needs to be informed by the Principal investigator not later than 15 days after abruption.

14 Literature

Introduction

1. Dietary Reference Intakes for energy, carbohydrates, fibre, fat, fatty acids, cholesterol, protein and amino acids. (2005) Institute of Medicine of the National Press.

2. Nutrition during Lactation (1991) Institute of Medicine National Academy Press.

3. Günther ALB, Buyken AE, Kroke A. The influence of habitual protein intake in early childhood on BMI and age at adiposity rebound: results from the DONALD Study. Int J Obesity (2006) 30: 1072-9.

4. Günther ALB, Buyken AE, Kroke A. Protein intake during the period of complementary feeding and early childhood and the association with body mass index and percentage body fat at 7 y of age. AJCN (2007) 85: 1626-33.

5. Arenz S, Rückerl R, Koletzko B, von Kries R. Breast-feeding and childhood obesity – a systematic review. Int J Obesity (2004) 28: 1247-56.

6. Koletzko B, von Kries R, et al. Lower protein in infant formula is associated with lower weight up to age two years: a randomised clinical trial. AJCN (2009) 89:1-10.

7. Fleischer Michaelsen K, Hoppe C, Molgaard C. Effect of early protein intake on linear growth velocity and development of adiposity. Monatsschr Kinderheilkd (2003) 151:S78-83.

8. Janas LM, Picciano MF, Hatch TF. Indices of protein metabolism in term infants fed human milk, whey-predominant formula, or cow's milk formula. Pediatrics (1985) 75:775-84.

9. Janas LM, Picciano MF, Hatch TF. Indices of protein metabolism in term infants fed either human milk or formulas with reduced protein concentration and various whey/casein ratios. J Pediatrics (1987) 10: 838-48.

10. Järvenpää AL, Räihä NCR, Rassin DK, Gaull GE. Milk protein quantity and quality in the term infant. I. Metabolic responses and effect on growth. Pediatrics (1982a) 70:214-20.

11. Järvenpää AL, Räihä NCR, Rassin DK, Gaull GE. Milk protein quantity and quality in the term infant. II. Effects on acidic and neutral amino acids. Pediatrics (1982b) 70:221-30

12. Räihä N, Minoli I, Moro G. Milk protein intake in the term infant. I. Metabolic responses and effects on growth. Acta Paediatr Scand (1986) 75: 881-6.

13. Ziegler E. Protein requirements in infancy. In: Infant formula: Closer to the reference. Ed. Räihä NCR, Rubltelli FF. Nestlé Nutrition Workshop Series Ped Program (2002) 47: S97-110.

14. Räihä NC, Fazzolari.Nesci A, Cajozzo C, Puccio G, Monestrier A et al. Whey predominant, whey modified infant formula with protein/energy ratio of 1.8g/100kcal: adequate and safe for term infants from birth to four months. J Ped Gastroent Nutr (2002) 35:275-81.

15. Turck D, Grillon C, Lachambre E, Robiliard P, Beck L et al. Adequacy and safety of an infant formula with a protein/energy ratio of 1.8g/100 kcal and enhanced protein efficiency for term infants during the first 4 months of life. J Ped Gastroent Nutr (2006) 43: 365-71.

16. Fomon SJ, Ziegler EE, Nelson SE, Frantz JA. What is the safe protein-energy ratio for infant formula. AJCN (1995) 62: 358-63.

17. COMMISSION DIRECTIVE 2006/141/EC of 22 December 2006 on infant formulae and follow-on formulae and amending Directive 1999/21/EC p. L 401/2.

18. ESPGHAN Committee on Nutrition. Guidelines on infant nutrition. I. Recommendations for the composition of an adapted formula. Acta Paediatr Scand Suppl (1977) 262:1-20.

19. Codex Alimentarius Commission. Joint FAO/WHO food standards programme. Codex Standard for infant formula (Codex-Stan72-1981) (1994) In: Codex Alimentarius, Vol 4, 2nd ed, FAO/WHO Rome.

20. Committee on Nutrition, American Academy of Pediatrics. Commentary on breast feeding and infant formulas, including proposed standards for formulas. Pediatrics (1976) 57: 278-85.

21. Koletzko B et al. The roles of long-chain polyunsaturated fatty acids in pregnancy, lactation, and infancy: review of current knowledge and consensus recommendations. JPM (2008) 36: 5-14.

22. Scientific Committee on Food. Report of the Scientific Committee on Food on the revision of essential requirements of infant formulae and follow-on formulae. SCF/CS/NUT/IF/65 Final Retrieved 1 mar 2006, from http://europa.eu.int/comm/food/fs/sc/scf/out199_en.pdf. 2003).

23. WHO Multicentre Growth Reference Study Group. WHO Child Growth Standards: Length/height-for-age, weight-for-age, weight-for-length, weight-for-height and body mass index-for-age: Methods and development. Geneva, World Health Organization (2006).

15 Appendices

15.1 Appendix: Formula composition: Study Formula

15.2 Appendix: Formula composition: Control Formula

15.3 Appendix: Instruction for Preparation; Dosage and Drinking amounts

15.4 Appendix: Study Formula Inventory Form

15.5 Appendix: Flow Chart

15.6 Appendix: Time Event Schedule

15.7 Appendix: Parent Information

15.8 Appendix: Informed Consent

15.9 Appendix: Subject Screening List

15.10 Appendix: Subject Identification List

15.11 Appendix: Randomisation List

15.12 Appendix: Case report Form (CRF)

15.13 Appendix: Diaries Formula and breastfeeding group

15.14 Appendix: Protocol of the reminder call

15.15 Appendix: Amino acid profiling

15.16 Appendix: Fatty acid profiling

15.17 Appendix: Material provided for blood withdrawal

15.18 Appendix: SOP for Blood – Sample Collection and Handling

15.19 Appendix: Serious adverse events form

15.20 Appendix: Declaration of Helsinki

15.21 Appendix: Monitoring SOP

15.22 Appendix: Protocol Violation Form

15.23 Appendix: Study Formula Random Number List

Formula with modified content of protein and improved fatty acids and their impact on infant growth and health Study nr: 502042

Amendment No.1

During the implementation of the study at the Clinical Centre Belgrade, it turned out that for two aspects the actual situation differs from assumptions on which the study protocol (Ver. 03.11.2009) is based. Thus, the following changes are required:

1. Rescheduling of the baseline visit

- Status quo: According to the study protocol the baseline visit is performed on postnatal days 4 or 5.
- Changes to study protocol: Instead of performing the baseline visit on postnatal days 4 or 5, the baseline visit is performed on postnatal days 3 or 4 (day of birth = postnatal day 0).
- Reason: Contrary to the initial assumption, most of the mothers, who delivered vaginally, leave the hospital already on postnatal day 3. Thus, these infants would be lost as potential study subjects and the study could not be completed on time.
- Background: During the first week of life biochemical parameters change rapidly, thus we aim for a narrow time window of sample collection. This narrow time window is maintained after rescheduling of the baseline visit.

2. Change of procedure for the enrollment of infants for the breast fed group in the course of the study

- Status quo: According to the study protocol infants are enrolled on postnatal days 3 or 4 independent of the parental intention to breast feed or not and 100 breast fed infants and 200 formula fed infants shall be studied (equal group sizes in the breast fed reference group and the two randomized formula groups)
- Amendment to study protocol: Once 100 breast fed infants have passed visit 1, no more infants will be enrolled for the breastfeeding group and no more breastfed infants will participate in visits 1 to 4.
- Reason: Contrary to the initial assumption the percentage of mothers who breast feed beyond day 28 is about 70%. Thus the predefined number of

subjects in the reference group (100 breast fed infants, who completed the study according to protocol) can be expected to be reached much earlier than the completion of the formula groups.

- Background: Infants have to be enrolled into the study on postnatal days 3 or 4 (baseline visit). But randomization into one of the formula groups can take place until postnatal day 28, if parents have meanwhile opted for formula feeding. Thus, it is unknown at time of enrollment, whether the infant will become eligible for one of the randomized formula groups. On the other hand, the time until the number of necessary subjects in the study groups is achieved may differ considerably between the groups. Considering the high percentage of breast feeding we expect the reference group to be completed much earlier. In case enrolled subjects are breastfed beyond postnatal day 28 they should not participate in study visits 1 to 4 once 100 infants have passed visit 1 in this group, furthermore no more subjects should be enrolled for the breastfeeding group. This would not be justified for ethical reasons and not possible for logistical and budgetary reasons.
- Practical implementation: At time of enrollment all parents will be informed that it is possible that the participation of their child will be terminated if the number of necessary subjects has already passed visit 1 in the corresponding study group. This will be documented in the case report form for visit 1 giving the reason: “excluded, because maximal participant number in the group meanwhile achieved”. The infants will not participate in visits 1, 2, 3 and 4, but they will not be designated drop outs, as it was not the decision of the families to terminate participation. Collection of the baseline data will allow to test, whether the infants studied until visit 4 are representative for all enrolled infants. Whenever 100 subjects have passed visit 1, no more subjects will be enrolled in the breastfeeding group.

Formula with modified content of protein and improved fatty acids and their impact on infant growth and health Study nr.: 502042

Amendment No.2

During the implementation of the study at the Clinical Centre of Serbia in Belgrade, it turned out that for the following aspect the actual situation differs from assumptions on which the study protocol (Ver. 03.11.2009) including Amendment version 1.1 is based. Thus, the following changes are made:

Omission of blood sampling at the baseline visit and changes regarding the window for performing the baseline visit

- Status quo: According to the study protocol a blood sample is taken at the baseline visit (postnatal days 3 to 4) to determine:
  - ✓ full blood count with differential, C-reactive protein, Astrup, electrolytes Na / K / Cl, Glucose, total bilirubin and protein
  - ✓ serum albumin, serum creatinine, blood urea nitrogen
  - ✓ plasma amino acid profile, plasma phospholipid fatty acid profile
- Change to study protocol: the blood sampling at baseline visit will be skipped. All other data assessments at the baseline visit will be maintained (see study protocol: chapter 8.5.1, page 36). The window for performing the visit is changed from postnatal days 3 to 4 to a wider window between birth and postnatal day 14 or before day 28 at the latest, if the investigator is sure that the subject is still able to shift to full formula feeding.
- Reason: For the success of the study 100 infants per group need to be enrolled (100 breast fed; 100 control formula fed and 100 modified formula fed). The enrollment is competitive which means if in one group 100 infants are enrolled no more subjects will be enrolled into this group. As the breast fed group is completed no more subjects are to be enrolled into this group.

  To reduce the number of blood withdrawals the blood draw on baseline visit is skipped as it is estimated that approx. 6 infants have to pass the baseline visit (including blood sampling) for each infant randomized into a formula group (problem of available resources and ethics). Thus, an adaption of the study protocol is required and is considered possible without sacrificing major scientific aims of the study. A comparison of the clinical data at baseline between the groups can be performed, which permits the exclusion of major differences between the groups. For the statistical analysis of biochemical parameters at study end the adjustment for baseline values obtained during the first postnatal days is not considered mandatory. A wider window for the

performance of the baseline visit does not cause a loss of data as the collected information is either independent of time or is obtained retrospectively from hospital records. On the other hand, this is expected to increase the participation rate.

- Practical implementation:
  - **Screening** for eligible formula-subjects is performed in the center by study team members on the maternity ward
    - → complete the „screening-list“
  - **Personal contact** with potential subjects: in case of interest
    - → check for inclusion and exclusion criteria
    - → verbal information for interested parents (duration, visits, blood sampling at the end of the study);
    - → hand over printed „parents information“ and “informed consent” / “accept form”
  - **in case of decision to participate in the study immediately after screening:**
    - → detailed explanation of the study
    - → after clarification of all open questions signature of informed consent (if parents decide not to sign this is recorded in the screening list and personal information is deleted)
    - → If subject is suitable to the study and in case the family wants to formula feed / is already feeding formula an appointment for the baseline visit should be made (parents need to be called / contacted for scheduling the baseline visit).
    - → randomization and distribution of study formula with corresponding randomization number
    - → complete “subject identification list” and “subject randomization list” (subject randomization number will be transferred from HiPP ambulance)
    - → performance of baseline visit without blood sampling, but otherwise according to original protocol
  - **in case parents wish to decide about participation later:**
    - → ask for contact data / telephone number (“accept form” to be filled out, signed by parents).
    - → inform parents that they will be called within the next week by a member of the study team
    - → do not perform baseline visit in the clinic

- → call parents and ask for interest within the first week after screening and, if they do not decide, again during the second week after screening (done by HiPP office, Belgrade)
- → if the family is not interested in participation, this is recorded in the screening list and all personal information is deleted, but otherwise no further action is required
- → only in case the family wants to formula feed / or does already feed formula and is interested in participation, the family is invited to the clinic for the baseline visit, an appointment is made and clinical personal is informed
- → before the baseline visit the study is explained in detail
- → after clarification of all open questions signature of informed consent (if parents decide not to sign this is recorded in the screening list and personal information is deleted)
- → randomization and distribution of study formula with corresponding randomization number
- → complete “subject identification list” and “subject randomization list”
- → performance of baseline visit without blood sampling, but otherwise according to original protocol

Formula with modified content of protein and improved fatty acids and their impact on infant growth and health

Study nr.: 502042

Amendment No.3

Status Quo

Aim of the BeMIM study was to provide a formula with a composition more similar to that of breast milk. The suitability of an infant formula with modified protein content and improved fatty acids for the particular nutritional use of healthy term infants was investigated against a standard formula (control). As hypothesized under the primary objective at the end of the intervention period there was no significant difference in weight gain between the control and the intervention group (data currently submitted to *Clinical Nutrition*). Based on these data it was concluded that the protein-reduced formula is suited for the exclusively feeding of healthy term infants.

Changes to study protocol:

Recently presented data show that a lower dietary protein intake in infancy might be positive for the weight development of children at an age of six years (6-years data of the CHOP study presented at the ESPGHAN congress 2013). For this reason a follow up of the BEMIM-Study is planned. This will include two additional visits around the 4th and 6th birthday of the children, who completed visit 1 of the BeMIM study. Aim is to investigate if the protein intake between 1-4 months has an impact on the weight development of children at an age of 4 and 6 years. The anthropometric measurements will be non-invasive to keep the burden of the children at a minimum. Therefore, the study duration shall be prolonged until the children reach an age of 6 years to evaluate that a protein-low diet in early life has an effect on the weight development of young children.

Reason:

Data of the CHOP study, presented at the ESPGHAN congress in 2013, suggest that the protein intake in infancy has an impact on body composition at 2 and 6 years of age. A

higher protein intake was associated with both increased muscle and fat deposition with a stronger effect on the latter. With the additional anthropometric measurements at an age of 4 and 6 years it will be tested if the observations of the CHOP study are valid in the BeMIM population.

The randomisation will not be broken as the clinical personal in Belgrade had no access to the list of random numbers. The list is kept by the sponsor, who provided the group assignment of the children to the scientific team at the Dr. von Hauner Children Hospital after the data analysis was completed.

Practical implementation:

Contact of parents:

The parents/caregivers of the children who participated in the BeMIM study will be contacted by phone or mail six weeks prior to the appointment (around the 4th or 6th birthday of the child). Parents will be informed about the objective and content of the follow-up visit and in case they are interested to participate, a parental pre-information letter will be send via mail. An additional reminder call will follow shortly before the follow-up visit.

At the appointment the identity of the children will be checked first. Afterwards a paediatrician will clarify any questions of the parents with regard to the study. Once all questions are clarified the parents will be asked to sign a written informed consent in the presence of the medical doctor.

Examination:

Clinical:

A paediatrician will conduct anthropometric measurements (body weight, body length, and head circumference) as described in chapter *8.5.1.3* of the study protocol. Additionally the body composition will be evaluated through a skin fold test and with a bioelectric impedance analysis (BIA).

Skin fold test will be conducted according to C. Brook (1971): Measurements will be made by the same paediatrician using a skin fold calliper performed at the triceps, biceps, subscapular, and suprailiac sites in each child.

BIA test will be made with a Nutrigard-MS apparatus according to manufacturer's instruction: The paediatrician will attach a gel electrode to the hand and foot of a child. The

measuring instrument is then connected to the electrodes and the measuring routine is started. The procedure will take approximately 2 minutes.

Questionnaire:

Additional to the clinical examination a small questionnaire with following questions need to be filled out:

Mother & father:

- Age
- Weight and length (BMI evaluation)
- Education (basic – additional – tertiary)
- Smoking

Additionally, the parents/caregivers will be asked to provide growth data of their children at an age of 2 and 3 years (if available).
